# Multiomics identifies a prognostic signature and SPIB as a potential regulator of gastric cancer lymph node metastasis

**DOI:** 10.1016/j.isci.2026.116774

**Published:** 2026-07-16

**Authors:** Zhijie Duan, Keyu He, Dianjie Chen, Lifan Lin, Ming Cao, Shaowei Zhang, Junbo Zhuang, Yintong Zhu, Guodong Shen, Yanfeng Hu

**Affiliations:** 1 Department of General Surgery, Guangdong Provincial Key Laboratory of Precision Medicine for Gastrointestinal Tumor, Nanfang Hospital, Southern Medical University, Guangzhou, Guangdong 510515, China; 2 Department of Surgery, Tongchuan Maternal and Child Health Care Hospital, Tongchuan, Shaanxi 727000, China; 3 The First School of Clinical Medicine, Nanfang Hospital, Southern Medical University, Guangzhou, Guangdong, China

**Keywords:** gastric cancer, lymph node metastasis, prognostic model, machine learning, tumor heterogeneity, biomarker

## Abstract

The heterogeneity within the same AJCC TNM stage of gastric cancer necessitates robust prognostic biomarkers beyond conventional staging. By integrating transcriptomic data from 1,613 patients across eight cohorts, we developed an 11-gene lymph node metastasis gene signature (LMGS) using an ensemble learning strategy to overcome the instability of traditional models. LMGS robustly predicted survival (mean C-index = 0.619), providing incremental prognostic value beyond conventional AJCC staging. Single-cell and spatial transcriptomic analyses revealed LMGS enrichment in epithelial/immune cells at the tumor-normal interface. Integrative analysis nominated the transcription factor SPIB as a candidate regulator, which was specifically overexpressed in metastatic lesions. Our study provides a clinically relevant prognostic tool and identifies SPIB as a molecular marker associated with lymphatic dissemination.

## Highlights

• Ensemble learning yields prognostic gastric cancer lymph-metastasis signature (LMGS)
• Eight cohorts (1,613 patients) validate prognostic performance of the 11-gene LMGS
• Multi-omics integration (Bulk, single-cell, and spatial) characterizes the LMGS
• SPIB is associated with lymph node metastasis in gastric cancer

## Graphical abstract

**Figure:**
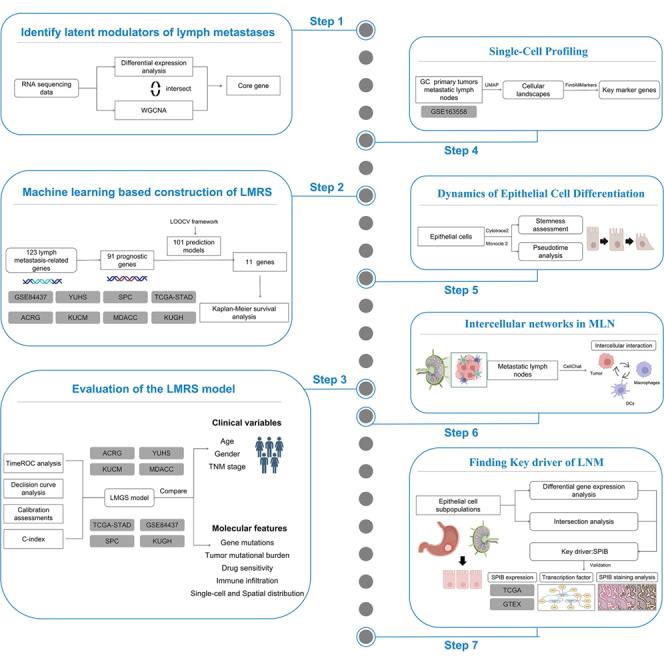

## Teaser

Bioinformatics; Cancer; Systems biology

## Introduction

Gastric cancer (GC) remains a major global health burden, ranking fifth in both incidence and mortality worldwide.^1^ Despite advances in standard treatment, the five-year survival rate for advanced GC remains below 30%.^2^^,^^3^ Inter- and intra-tumoral heterogeneity leads to markedly divergent outcomes even among patients with the same stage, thereby limiting the prognostic accuracy of the AJCC TNM system.^4^ Lymph node metastasis (LNMs) is a critical driver of GC progression and a well-established sign of poor prognosis, as nodal involvement both reflects enhanced invasiveness and facilitates systemic dissemination.^5^^,^^6^^,^^7^^,^^8^^,^^9^ Bulk RNA sequencing has revealed key metastatic gene expression signatures and yielded candidate targets in lymph-metastatic GC,^10^^,^^11^^,^^12^ but inherently masks cell-type-specific dynamics. Single-cell RNA sequencing (scRNA-seq) overcomes this limitation by resolving tumor cell heterogeneity and microenvironment interactions at high resolution,^13^^,^^14^^,^^15^ delineating malignant epithelial subpopulations and immune-stromal crosstalk in GC metastases.^16^^,^^17^ Yet spatial transcriptomics preserves tissue localization to map this information to anatomical niches, and few studies have systematically integrated scRNA-seq and spatial transcriptomics with bulk RNA-derived prognostic models, limiting their ability to both stratify patient risk and identify metastasis-associated genes. Accordingly, there is an urgent need for a robust, molecularly informed prognostic model capable of accurately stratifying patients and guiding personalized therapies. In particular, uncovering key molecular markers of lymphatic dissemination within malignant epithelial cells is also essential for understanding the mechanisms of this process.

Some bioinformatic efforts incorporating LNM-associated genes such as EMT markers into prognostic models have improved survival stratification in patients with GC,^18^^,^^19^ but these models predominantly employ LASSO regression, which (1) selects only one predictor from highly correlated gene sets and (2) requires precise tuning of its penalty parameter (λ), thereby limiting robustness and generalizability.^20^^,^^21^^,^^22^^,^^23^^,^^24^^,^^25^ In order to address these shortcomings of traditional prognostic models, the leave-one-out cross-validation (LOOCV) framework has been shown to be a robust alternative, providing enhanced stability and predictive power in high-dimensional settings.^26^^,^^27^ However, it has not systematically fused bulk-derived prognostic modeling with single-cell and spatial dissection to both stratify patient risk based on LNM biology and identify metastasis-driving genes in specific cell compartments.

Here, we constructed and validated an ensemble-based LNM-related gene signature (LMGS) across eight independent cohorts (1,613 patients) using LOOCV. Molecular biomarkers of lymphatic dissemination were further identified, and SPIB was nominated as a potential regulator.

## Results

### Identification of differentially expressed genes and key gene modules

**Figure 1:**
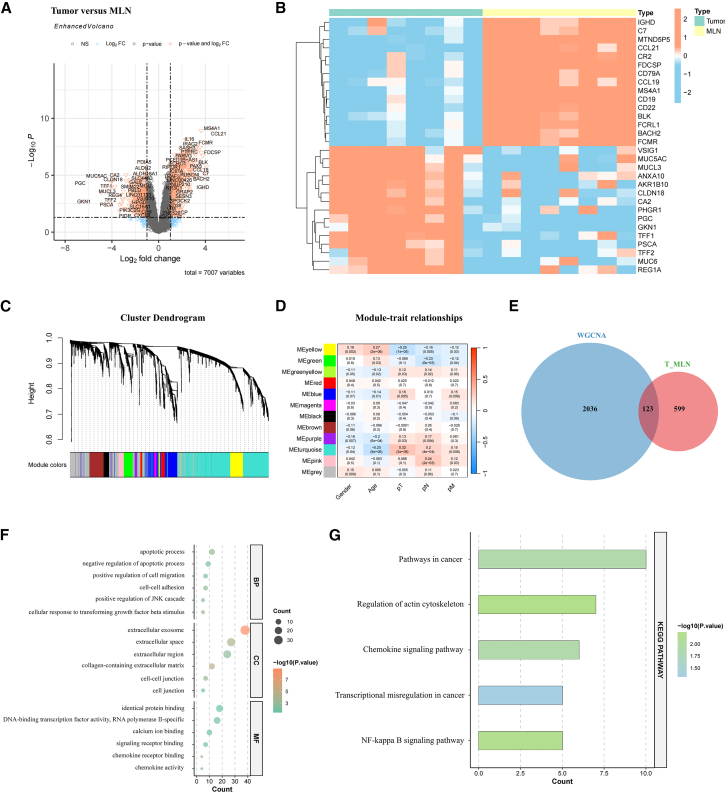
Identification of lymph metastases-related genes and functional enrichment analysis (A) Volcano plot shows differentially expressed genes between primary GC and metastatic lymph nodes. Orange: upregulated genes; Blue: downregulated genes; Gray: non-significant genes. (B) Heatmap depicts the top 15 DEGs across paired samples. Red indicates tumor samples; blue indicates metastatic lymph nodes. Genes were clustered based on *Z* score normalization. (C) Cluster dendrogram of gene modules identified by WGCNA in the ACRG cohort. Hierarchical clustering of genes based on co-expression patterns, with different colors representing distinct gene modules. (D) Correlation analysis between module eigengenes and clinical traits. (E) The overlapping Genes between WGCNA(left blue) and DEGs(right green). (F) GO enrichment analysis of overlapping genes between WGCNA and DEGs, displayed as a bubble plot. (G) KEGG pathway enrichment analysis of overlapping genes between WGCNA and DEGs, shown as a bar plot.

To pinpoint critical molecular regulators of LNM in GC, we performed bulk RNA sequencing on paired primary tumor tissues and matched metastatic lymph nodes. Differential expression analysis identified 384 significantly upregulated and 338 significantly downregulated genes between the two tissue types (|log2 fold change| > 1, *p* < 0.05) (Figure 1A). The top 50 differentially expressed genes further highlighted distinct transcriptional profiles between primary and metastatic samples (Figure 1B). Next, weighted gene co-expression network analysis (WGCNA) was conducted using the ACRG GC RNA-seq dataset (GSE62254) to characterize co-regulated gene modules associated with metastasis. Several modules were identified, with the blue and turquoise modules showing the strongest correlations with LNM (Figures 1C and 1D). We then refined our gene list by intersecting the upregulated DEGs with the metastasis-associated WGCNA modules, yielding a core set of 123 genes highly relevant to the metastatic cascade (Figure 1E).

Subsequent gene ontology (GO) and Kyoto Encyclopedia of Genes and Genomes (KEGGs) enrichment analyses elucidated the biological functions of these genes. GO terms were significantly enriched for processes such as cell adhesion, extracellular matrix (ECM) organization, and epithelial cell migration. KEGG pathway analysis revealed involvement in focal adhesion, ECM-receptor interaction, and TGF-β signaling pathways (Figures 1F and 1G), all of which play crucial roles in tumor invasion and metastasis. Together, these findings delineate a core gene signature associated with LNM in GC, implicating cellular adhesion and ECM remodeling as pivotal factors underlying metastatic progression.

### Consensus signature construction via multi-algorithm machine learning integration

**Figure 2:**
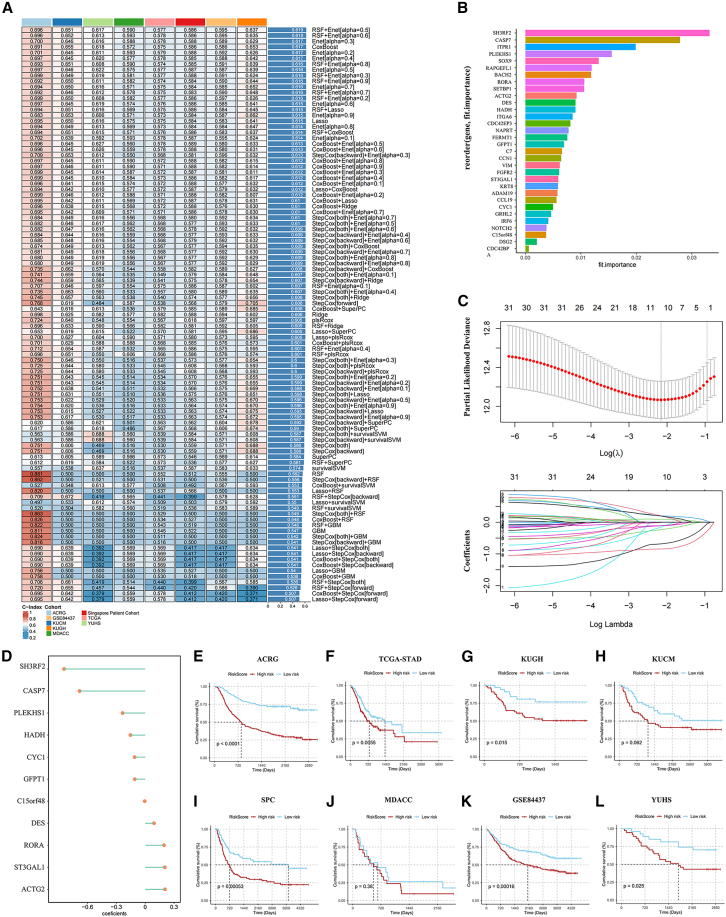
Development and validation of the consensus lymph metastasis-related gene signature (LMGS) using machine learning (A) A total of 101 prediction models were developed using the leave-one-out cross-validation (LOOCV) framework, and the C-index was calculated for each model across all validation datasets. (B) The most influential variables obtained from the random survival forest (RSF) algorithm in the ACRG cohort. (C) The best-fit λ was determined when the partial likelihood deviance reached its minimum value, leading to the generation of Enet coefficients for the most relevant prognostic genes. Data are presented as mean ± 95% confidence interval (CI). (D) Coefficients of 11 genes identified using Enet (α = 0.5). (E–L) Kaplan-Meier survival curves based on LMGS in different cohorts: (E) ACRG (log rank test: *p* < 0.0001), (F) TCGA-STAD (log rank test: *p* = 0.0055), (G) KUGH (log rank test: *p* = 0.015), (H) KUCM (log rank test: *p* = 0.062), (I) SPC (log rank test: *p* = 0.00053), (J) MDACC (log rank test: *p* = 0.38), (K) GSE84437 (log rank test: *p* = 0.00016), (L) YUHS (log rank test: *p* = 0.025).

We aimed to develop a robust LMGS for GC prognosis by initially identifying 91 prognostic genes significantly associated with overall survival from the expression profiles of 123 lymph metastasis-related genes using univariate Cox regression (*p* < 0.05). Integrative machine learning analysis was subsequently performed employing 101 distinct predictive algorithm combinations. To ensure model stability and generalizability across multiple datasets, a LOOCV framework was implemented. Among all tested models, the combination of random survival forest (RSF) and elastic net (Enet, α = 0.5) achieved the highest predictive performance, with a mean concordance index (C-index) of 0.619 across all validation cohorts (Figure 2A).

The best-fit regularization parameter (λ) for Enet regression was determined based on the minimum partial likelihood deviance (Figure 2C), following the initial RSF analysis (Figure 2B). Eleven genes with nonzero coefficients were retained, constituting the final prognostic gene panel exhibiting the strongest prognostic significance (Figure 2D).A risk score was then calculated for each patient as a weighted sum of the expression values of these 11 genes (SH3RF2, CASP7, PLEKHS1, HADH, CYC1, GFPT1, C15orf48, DES, RORA, ST3GAL1, ACTG2), with weights corresponding to their Cox regression coefficients. To ensure clinical reproducibility, the median prognostic index (PI) of the training cohort was established as the pre-specified fixed threshold (Cutoff = −0.0649505). Based on this training-defined cutoff, patients across all cohorts were stratified into high- and low-risk groups. Kaplan-Meier (KM) analysis demonstrated that this stratification effectively captured significant survival divergence in the majority of datasets (Figures 2E and 2L). Univariate Cox regression (Figure S1A) further validated the prognostic robustness of the LMGS as a continuous variable, showing significant associations with poorer OS in all cohorts (*p* < 0.05). In two small-sample cohorts where the survival analysis lacked statistical significance(MDACC, *p* = 0.38; KUCM, *p* = 0.062), the continuous model consistently supported the prognostic impact of the LMGS. These findings collectively suggest that the LMGS is a stable, potentially clinically deployable indicator independent of cohort-specific distributions.

### Evaluation of the LMGS model

**Figure 3:**
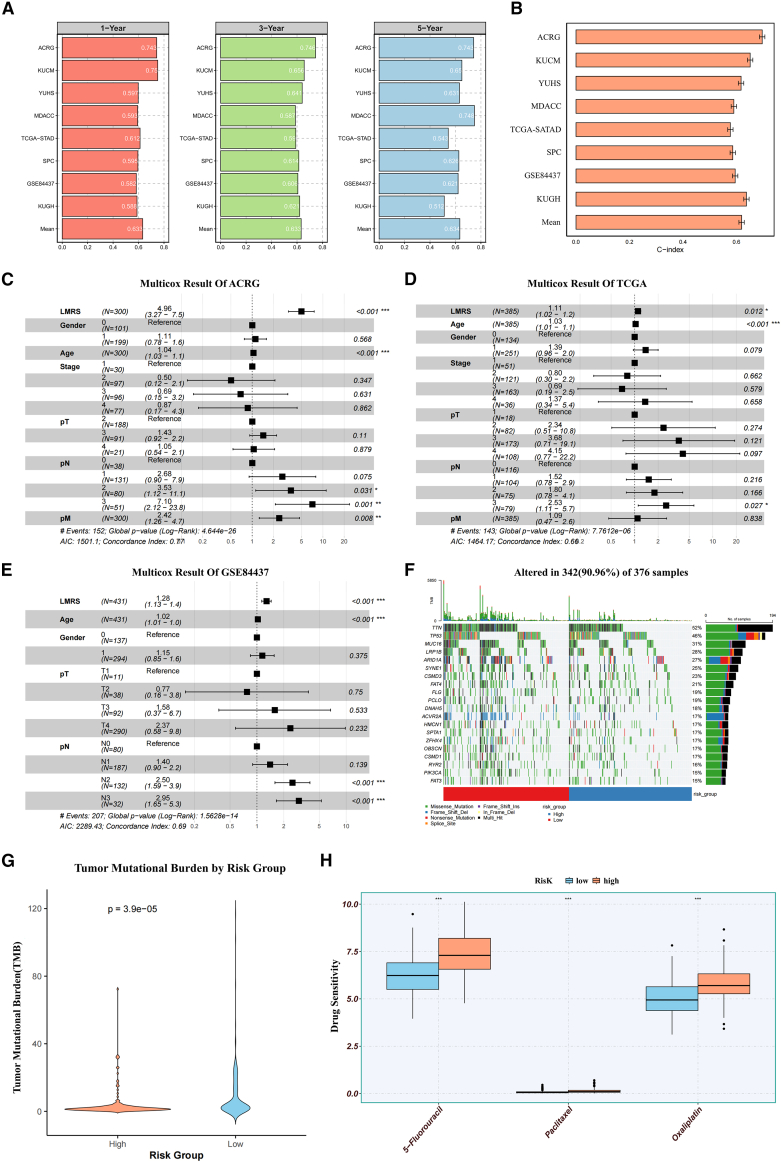
Evaluation of the LMGS model (A) Time-dependent ROC analysis for predicting OS at 1, 3, and 5 years. (B) C-index of LMGS across all datasets. (C–E) The performance of LMGS was compared with other clinical variables in predicting prognosis using multivariate Cox regression analysis in ACRG, TCGA-STAD, and GSE84437 cohorts. Hazard ratios (HR) with 95% confidence intervals (CI) are shown. Statistical test: two-sided Wald test. Data are presented as mean ± 95% CI. ∗*p* < 0.05; ∗∗*p* < 0.01; ∗∗∗*p* < 0.001; and ∗∗∗∗*p* < 0.0001. (F) Oncoplot of gene mutations in the TCGA cohort stratified by LMGS. Samples are ranked by LMGS score, with different colors representing distinct mutation types. (G) Tumor mutational burden in high and low LMGS groups in the TCGA cohort. Statistical test: two-sided *t* test. (H) Drug sensitivity of 5-FU, paclitaxel, and oxaliplatin in high and low LMGS groups across all cohorts.

The discriminative performance of the LMGS model was first evaluated using time-dependent receiver operating characteristic (timeROC) analysis, yielding area under the curve (AUC) values for 1-, 3-, and 5-year overall survival as follows: 0.7427, 0.7455, and 0.7430 in the ACRG cohort; 0.7504, 0.6563, and 0.6503 in KUCM; 0.5974, 0.6408, and 0.6309 in YUHS; 0.5929, 0.5865, and 0.7483 in MDACC; 0.6118, 0.5900, and 0.5431 in TCGA-STAD; 0.5949, 0.6140, and 0.6263 in SPC; 0.5821, 0.6064, and 0.6205 in GSE84437; and 0.5879, 0.6206, and 0.5118 in KUGH. The mean AUC across cohorts for 1-, 3-, and 5-year survival was 0.617, 0.644, and 0.625, respectively (Figure 3A).To further evaluate the model’s overall predictive performance, we calculated the C-index, which integrates information across all survival times. The C-index values [95% confidence interval] were 0.696 [0.662–0.730] for ACRG; 0.651 [0.612–0.690] for KUCM; 0.617 [0.578–0.656] for YUHS; 0.590 [0.540–0.640] for MDACC; 0.577 [0.522–0.633] for TCGA-STAD; 0.586 [0.544–0.628] for SPC; 0.595 [0.547–0.643] for GSE84437; and 0.637 [0.595–0.679] for KUGH, with a mean C-index of 0.619 across cohorts, further supporting the model’s robustness (Figure 3B). Finally, multivariate Cox regression analysis was performed to confirm the independent prognostic value of the LMGS. The results demonstrated that the LMGS remained a significant independent prognostic factor after adjusting for conventional clinical variables, including age, gender, and AJCC stage (Figures 3C and 3E). In summary, these results indicate the accuracy and robustness of our model.

To further substantiate the robustness and clinical utility of the LMGS, we first evaluated its predictive accuracy through calibration assessment across all eight independent cohorts. Calibration plots (Figure S1B) exhibited excellent agreement between the predicted survival probabilities and actual observed survival, confirming the high accuracy and robust generalizability of the LMGS signature. Building on this predictive reliability, we then performed decision curve analysis (DCA) to quantify the incremental clinical net benefit of the LMGS. Specifically, we constructed integrated models combining the LMGS with clinical parameters (AJCC stage, or pT plus pN indicators for the GSE84437 cohort where composite staging was unavailable). The DCA results across all validation sets revealed that the integrated model consistently provided a substantially higher clinical net benefit than clinical parameters alone (Figure S2). These findings confirm that the LMGS captures biological heterogeneity beyond traditional clinical classifications and provides significant incremental prognostic value.

### Comprehensive immunological and chemotherapeutic profiling

Given that therapeutic responses, including chemotherapy and immunotherapy, are major determinants of patient prognosis, we next evaluated the predicted immunological landscape and potential trends in treatment sensitivity associated with LMGS-defined risk groups. Immune infiltration analysis, performed using the IOBR R package, revealed significantly elevated levels of immune effector cells, including CD8^+^ T cells and natural killer (NK) cells, in the low-risk group (*p* < 0.001). Conversely, immunosuppressive cells such as cancer-associated fibroblasts (CAFs), M2 macrophages, and regulatory T cells (Tregs) were significantly enriched in the high-risk group (*p* < 0.001), suggesting a more immunosuppressive tumor microenvironment. Consistent with these observations, immunotherapy-related indices, such as the Immunophenoscore (IPS) and MHC-related scores, were significantly higher in the low-risk group (*p* = 0.002 and *p* = 0.004, respectively), suggesting a potential trend toward increased immunotherapy responsiveness(Figure S3). Somatic mutation analysis using the maftools R package in the TCGA-STAD cohort identified frequently mutated genes, including TTN, TP53, and MUC16. A broad spectrum of mutation types was observed, such as missense mutations, frameshift deletions, and nonsense mutations. Notably, the low-risk group exhibited a significantly higher tumor mutation burden compared to the high-risk group (*p* < 0.001) (Figures 3F and 3G).

In silico drug sensitivity analysis of commonly used GC chemotherapy agents, including 5-fluorouracil (5-FU), paclitaxel, and oxaliplatin, demonstrated significantly greater predicted sensitivity in the low-risk group (*p* < 0.001 for all) (Figure 3H). Taken together, these computational findings suggest that the low-risk group may possess a more favorable immune landscape, thereby potentially providing preliminary insights into increased responsiveness to both immunotherapy and conventional chemotherapy.

### Single-cell profiling of gastric cancer primary tumors and metastatic lymph nodes

**Figure 4:**
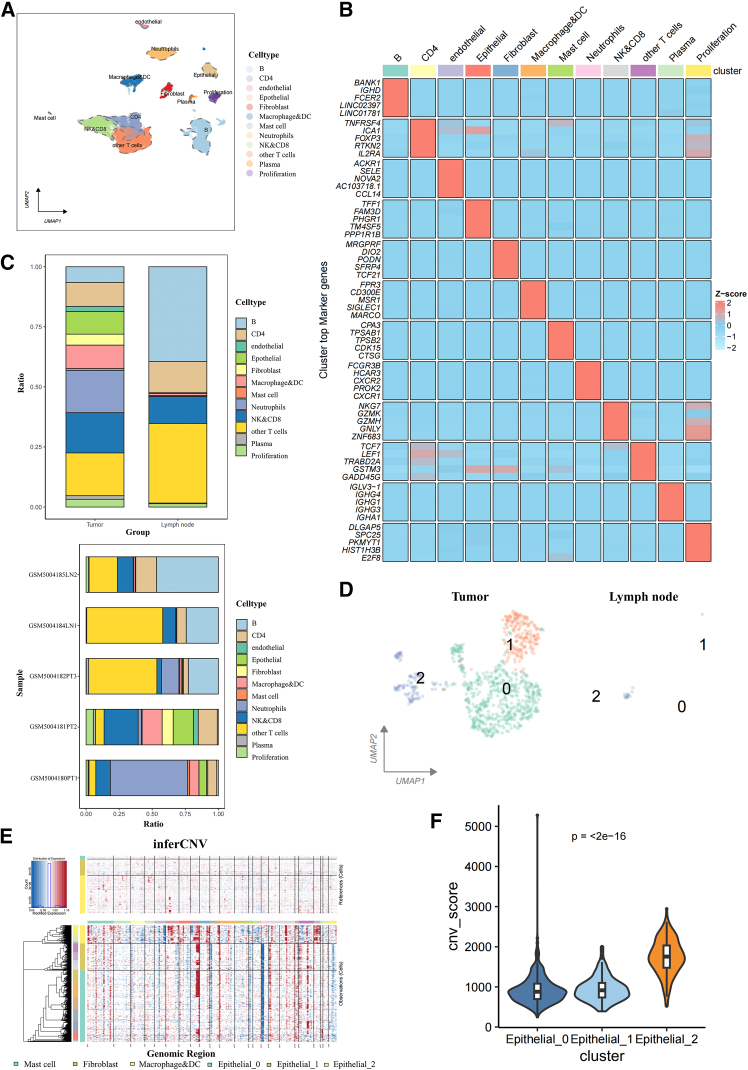
Single-cell transcriptome profiles of GC primary tumors and metastatic lymph node samples (A) UMAP plot showing the annotation and color codes for all cell types in primary gastric tumors and metastatic lymph nodes. (B) Bar plot depicts the relative proportions of different cell types in primary and metastatic samples across patients. (C) Heatmap of top 5 marker genes for 12 major cell lineages. (D) UMAP of Epithelial cells in all samples. (E) Heatmap shows the chromosomal distribution of large-scale copy number variations (CNVs) in epithelial cells, inferred from scRNA-seq. (F) CNV scores of epithelial cells.

To elucidate key factors involved in LNM of GC, we performed a comprehensive scRNA-seq analysis using publicly available data from the GEO database (GSE163558). This dataset comprised matched primary tumor tissues and metastatic lymph nodes. Dimensionality reduction via Uniform Manifold Approximation and Projection (UMAP) allowed visualization of distinct cellular landscapes across these tissue types (Figure 4A). We identified multiple major cell populations, including B cells, CD4^+^ T cells, endothelial cells, epithelial cells, fibroblasts, macrophages and dendritic cells (DCs), mast cells, neutrophils, NK and CD8^+^ T cells, other T cell subsets, plasma cells, and proliferating cells.

Immune cells constituted the largest proportion of cells, especially within metastatic lymph nodes, which may partially reflect differences in dissociation efficiency across cell types76, 77. Moreover, cell-type proportions varied between patient samples and tissue origins, underscoring the heterogeneity of the GC microenvironment (Figure 4B). To further characterize these populations, we analyzed expression profiles of key marker genes across all identified clusters using Seurat’s Find All Markers function. The resulting heatmap (Figure 4C) revealed distinct molecular signatures for each cell population, providing insights into the cellular architecture driving GC metastasis.

### Identification of malignant epithelial cells and CNV profiling

Focusing on epithelial cells to identify malignant tumor populations within GC samples, we extracted and re-clustered these cells for in-depth characterization. UMAP visualization revealed three distinct epithelial subpopulations, designated Epithelial_0, Epithelial_1, and Epithelial_2. Notably, the Epithelial_2 cluster was predominantly localized in metastatic lymph nodes (Figure 4D), suggesting its specific enrichment at metastatic sites.

We performed copy number variation (CNV) analysis using the InferCNV R package to identify malignant cells. This approach detects large-scale genomic alterations indicative of malignancy. CNV profiles calculated for each epithelial subpopulation demonstrated that Epithelial_2 harbored the highest CNV burden, particularly within metastatic lymph nodes (Figures 4E and 4F). Given that epithelial cells are typically absent in normal lymph nodes, the aberrant presence of the Epithelial_2 population in these tissues, combined with its elevated CNV burden, strongly indicates that this cluster represents malignant cells. These findings highlight significant genomic instability and cellular heterogeneity within the epithelial compartment of GC, emphasizing the role of the Epithelial_2 subpopulation in LNM.

### Dynamics of epithelial cell differentiation

**Figure 5:**
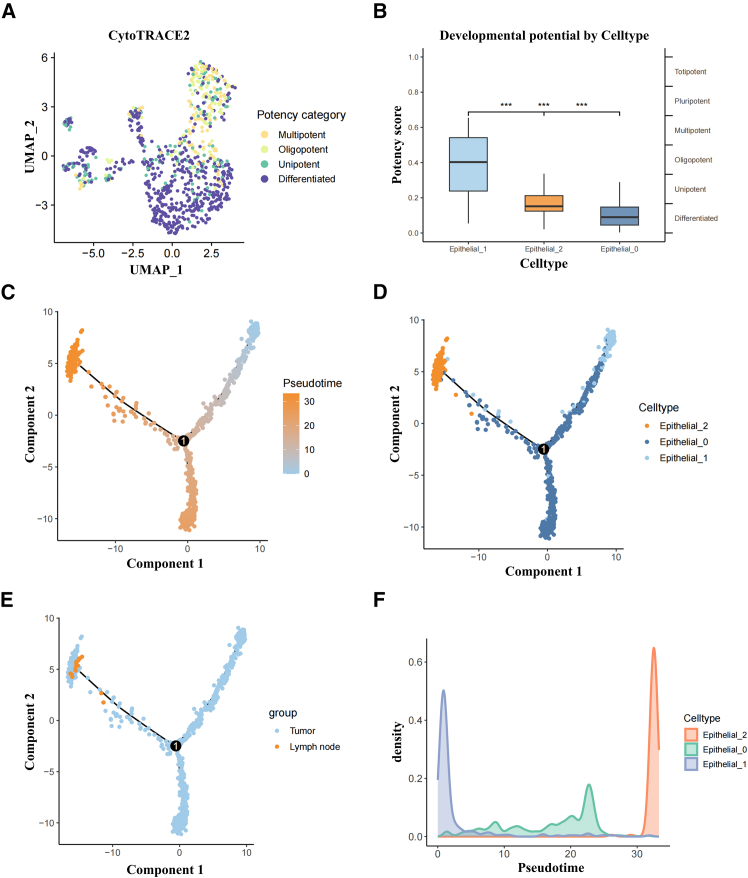
Developmental trajectory and evolution of epithelial cells in gastric cancer progression (A) UMAP embeddings of Epithelial cells. Plots are colored by CytoTRACE 2 potency category. (B) Potency stemness scores of 3 epithelial cell clusters using CytoTRACE2. (C) Pseudo time trajectory, with color gradient indicating progression from early (blue) to late (orange) stages. (D) Same trajectory, colored by epithelial subtypes (Epithelial_0, Epithelial_1, and Epithelial_2). (E) Trajectory with cells labeled by sample origin (tumor vs. metastatic lymph node). (F) Density plot shows pseudo time distribution across epithelial subtypes.

We first assessed epithelial subpopulation stemness using CytoTRACE2 to explore their developmental trajectories during GC progression. The UMAP plot (Figure 5A) classified epithelial cells into four distinct groups: multipotent, oligopotent, unipotent, and differentiated. The bar plot (Figure 5B) illustrates the potency scores, identifying Epithelial_1 as the most differentiated subpopulation, which was designated as the developmental starting point. Subsequently, Monocle 2 was applied to perform pseudo time analysis, enabling the reconstruction of epithelial cell differentiation dynamics. The unsupervised transcriptional trajectory and evolutionary density plots (Figures 5C and 5F) revealed a progression from the benign, differentiated Epithelial_1 subpopulation through an intermediate Epithelial_0 state, culminating in the malignant Epithelial_2 population predominantly found in metastatic lymph nodes. These analyses elucidate dynamic transitions across differentiation states, underscoring the malignant reprogramming accompanying LNM.

### LMGS activity at single-cell and spatial resolution

**Figure 6:**
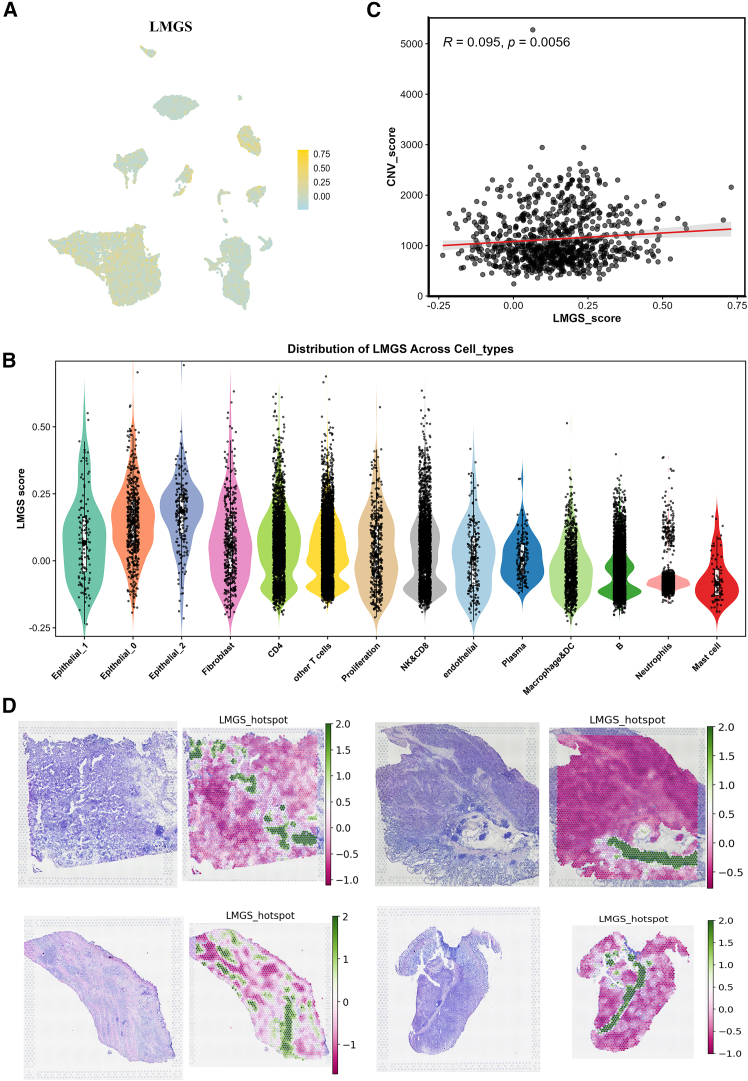
Spatial and single-cell distribution of LMGS activity in gastric cancer (A) Single-cell UMAP feature plots show the expression of individual LMGS genes across annotated cell types. (B) Violin plots of per-cell LMGS scores across all populations. (C) Scatterplot shows the relationship between per-cell LMGS scores and inferred CNV burden(Spearman’s ρ = 0.095; *p* = 0.0056). (D) Spatial map of LMGS hotspot in gastric cancer tissue.

To assess the cellular distribution of the prognostic LMGS, we examined the individual expression patterns of each gene(‘C15orf48' was not detected) across annotated cell types and found that LMGS-related transcripts were predominantly enriched in epithelial cells and specific immune subsets (Figure S4A). Per-cell LMGS scores were then computed using Seurat’s Add Module Score function. LMGS activity was largely concentrated in the metastatic malignant epithelial cluster (Epithelial_2), whereas other epithelial subclusters and immune populations displayed comparatively lower scores (Figures 6A and 6B). This distribution suggests potential associations between epithelial and immune compartments in tumor progression. Moreover, per-cell LMGS scores positively correlated with the inferred CNV burden (Spearman’s ρ = 0.095; *p* = 0.0056), reinforcing its association with malignant genomic alterations (Figure 6C).

To characterize the spatial organization of the LMGS signature, we analyzed a publicly available spatial transcriptomics dataset and identified eight independent molecular niches (Figures S4B and S4C). Given that the expression of individual genes alone cannot fully capture the spatially organized transcriptional programs within the tumor microenvironment, the Hotspot algorithm was employed to characterize the spatial co-expression architecture of the LMGS signature across the tissue. This analysis revealed markedly elevated LMGS intensity at the tumor-normal transition zone compared with intertumoral or adjacent normal regions across the majority of samples (Figure 6D). Collectively, integrated single-cell and spatial analyses demonstrate that LMGS activity concentrates within malignant epithelial compartments and exhibits spatially coordinated activation along the tumor-normal interface, suggesting a distinct molecular niche associated with tumor invasion and metastasis, with potential epithelial-immune crosstalk.

### Cell-cell communication networks in metastatic lymph nodes

**Figure 7:**
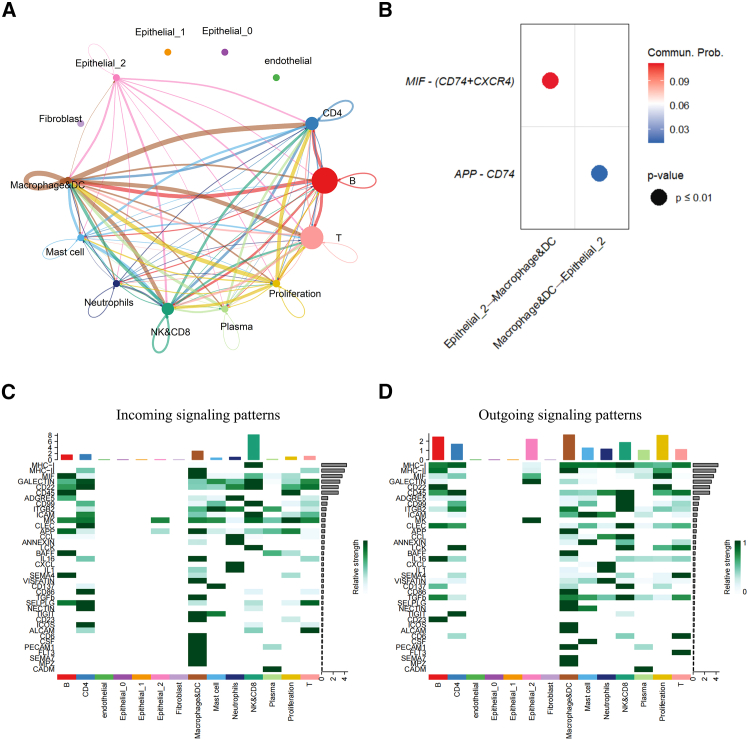
Intercellular networks in metastatic lymph nodes (MLN) (A) Circle plot depicts the cellular crosstalk among different cell types and subclusters; dot size and edge width indicate communication probability. (B) Bubble plot displays predicted ligand-receptor interactions that are uniquely significant in epithelial cells. (C and D) Heatmaps show the outgoing (C) and incoming (D) signal strengths of activated signaling pathways across all cell types in MLN; the color gradient reflects the mean interaction strength.

Intercellular interactions within the tumor microenvironment play a critical role in cancer progression and LNM, and we therefore analyzed cell-cell communication networks in metastatic lymph nodes using the Cell Chat R package. The comprehensive communication network highlighted the complex intercellular interactions present in this metastatic niche (Figure 7A). We specifically investigated the communication dynamics involving tumor cells (Epithelial_2) within the microenvironment. Notably, Epithelial_2 cells predominantly engaged in autocrine signaling as well as interactions with macrophages and DCs, mediated primarily via the MIF-(CD74 + CXCR4) and APP-CD74 ligand-receptor pairs, respectively (Figure 7B). Further analysis identified macrophages and DCs as the most active receptor-ligand interactors, underscoring their central roles within the metastatic milieu. As signaling sources, Epithelial_2 cells prominently activated GALECTIN, MDK, and MIF pathways, while also acting as receptors for signals originating from midkine (MK) and APP ligands (Figures 7C and 7D). These findings highlight the pivotal role of tumor cells in orchestrating cross-talk with immune populations and reveal key signaling pathways that may drive tumor progression and metastasis.

### SPIB as a potential molecular regulator in tumor cell migration and lymph node metastasis in gastric cancer

**Figure 8:**
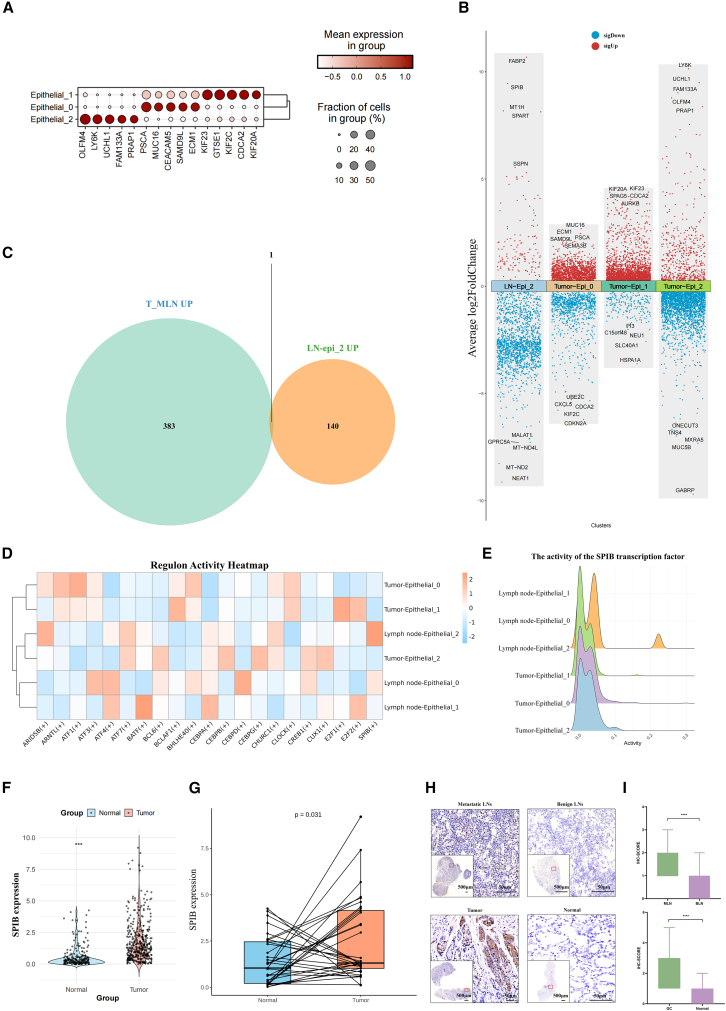
SPIB is a potential regulator in lymph node metastasis (A) Dot plot show the top 5 marker genes for the three epithelial subclusters. (B) Heatmap depicts upregulated and downregulated genes across all epithelial subclusters from primary tumors (PT) and metastatic lymph nodes (MLN). (C) Venn diagram shows SPIB as the only overlapping gene between MLN-upregulated genes (383 genes, left blue) and LN-Epi_2-upregulated genes (140 genes, right green). (D) Heatmap of regulatory factor activity scores (RFAS) calculated by pySCENIC for each epithelial cell cluster across PT and MLN. (E) Ridge plot shows SPIB regulatory factor activity score (RFAS) in each epithelial cell cluster across PT and MLN. (F) Analysis of SPIB expression between tumor and normal groups in the TCGA-STAD and GTEX datasets. Statistical test: two-sided unpaired *t* test. Data are presented as mean ± 95% CI. (G) Paired analysis of SPIB expression between tumor and adjacent normal tissues in the TCGA-STAD cohort. Statistical test: two-sided paired *t* test. Data are presented as mean ± 95% CI. (H) Representative IHC staining images of SPIB in tumor tissues, adjacent normal tissues, benign lymph nodes, and MLN (*n* = 20). Scale bars, 500 μm, 50 μm. (I) Analysis of IHC scores between tumor tissues, adjacent normal tissues, benign lymph nodes, and MLN based on SPIB staining results (*n* = 20, *p* < 0.0001).

To identify key molecular regulators of LNM in GC, we performed differential gene expression analysis comparing general epithelial subpopulations (Epi_0–2) with epithelial subsets from primary tumors and metastatic lymph nodes (Tumor-Epi_0–2, LN-Epi_0–2) (Figures 8A and 8B). Subpopulations with fewer than three cells (LN-Epi_0–1) were excluded from the analysis. Intersection analysis (Figure 8C) revealed shared upregulated genes between metastatic lymph node tumor cells (LN-Epi_2) and upregulated genes from bulk transcriptomic data of metastatic lymph node tissues. Notably, SPIB was the sole overlapping gene. Additionally, transcription factor activity was assessed using the SCENIC framework, revealing significantly elevated SPIB-associated activity in tumor cells from metastatic lesions relative to other epithelial subpopulations (Figures 8D and 8E). Further validation was conducted by analyzing SPIB expression in TCGA and GTEX datasets, where SPIB was significantly upregulated in tumor samples relative to adjacent normal tissues (Figure 8F). Paired sample analysis of the TCGA-STAD cohort (Figure 8G) confirmed markedly higher SPIB expression in tumor tissues compared to matched normal controls. This finding was corroborated by immunohistochemical (IHC) staining of pathological sections from our center, showing significantly elevated SPIB expression in both tumor tissues and metastatic lymph nodes (Figures 8H and 8I). Altogether, these results suggest the potential regulatory importance of SPIB in GC metastasis, suggesting its potential as a candidate biomarker for future functional and clinical investigation.

## Discussion

The AJCC TNM staging system shows substantial prognostic heterogeneity, as patients within the same stage can experience markedly different outcomes.^4^ LNM is a major contributor to this variation and correlates with poor survival in GC.^5^^,^^9^ Previous LNM-based prognostic models using LASSO regression have often been limited by feature instability and the neglect of correlated biomarkers.^22^^,^^23^^,^^24^^,^^25^ Here, we developed and validated an 11-gene LMGS via ensemble learning with LOOCV across bulk transcriptomes from 1,613 patients with GC in eight cohorts. LMGS robustly stratified overall survival and provided incremental prognostic value beyond conventional AJCC staging.

Effective management of GC relies on understanding the interplay between tumor genomics, immune contexture, and chemotherapeutic sensitivity. High tumor mutational burden (TMB) has been linked to improved immunotherapy outcomes through enhanced neoantigen presentation and T cell activation,^28^^,^^29^^,^^30^ while a balance between effector and suppressive immune populations within the tumor microenvironment influences clinical response.^31^^,^^32^^,^^33^ Moreover, patient benefit from frontline chemotherapies such as 5-FU, paclitaxel, and oxaliplatin varies widely. In our study, the LMGS low-risk group demonstrated significantly higher TMB (consistent with previous studies showing better prognosis in high-TMB GC) and frequent mutations in TTN, TP53, and MUC16.^34^^,^^35^^,^^36^^,^^37^ Immune deconvolution further revealed that low-risk tumors were enriched for CD8^+^ T cells, activated CD4^+^ T cells, plasma cells, and M1 macrophages with elevated MHC expression, whereas high-risk tumors harbored Tregs, M2 macrophages, myeloid-derived suppressor cells, and cancer-associated fibroblasts, typifying an immunosuppressive niche.^38^^,^^39^^,^^40^^,^^41^^,^^42^ Finally, in silico drug sensitivity profiling suggested that low-risk patients might exhibit better predicted responses to standard chemotherapeutics, highlighting the potential of the LMGS to stratify patients for both immunotherapy and chemotherapy. Looking forward, the 11-gene LMGS could be developed into a clinical RT-qPCR assay kit to refine prognostic assessment. By complementing TNM staging, this tool would offer a molecular basis for risk stratification, helping to tailor individual surveillance and treatment strategies in GC.

Bulk RNA-seq analyses have uncovered gene expression patterns linked to metastatic progression but mask the cellular heterogeneity driving metastasis.^13^^,^^14^^,^^15^ To resolve this, we analyzed publicly available single-cell RNA-seq data from matched primary tumors and metastatic lymph nodes, uncovering extensive remodeling of the metastatic niche. Within the epithelial compartment, we identified three transcriptionally distinct subpopulations, among which Epithelial_2 was highly enriched in lymph nodes and exhibited significant copy-number variations indicative of malignancy. Pseudo time trajectory inference mapped a continuum from benign-like to malignant epithelial states, underscoring dynamic epithelial plasticity during lymphatic dissemination. We also observed that LMGS activity is enriched in malignant epithelial cells and specific immune subsets, and correlates with CNV burden, linking it to malignant genomic alterations. Spatially, LMGS exhibits coordinated activation at the tumor-normal interface, defining discrete molecular niches. These findings suggest that LMGS reflects both cell-intrinsic malignant states and spatially organized transcriptional programs, potentially contributing to tumor invasion and progression.

Cell-cell communication analysis via Cell Chat revealed that Epithelial_2 cells engage in autocrine and paracrine signaling with macrophages and DCs through the MIF-(CD74 + CXCR4) and APP-CD74 axes, with the MK pathway serving as a central mediator of pro-metastatic immune crosstalk.^43^ By integrating the differentially expressed genes identified from our bulk RNA-seq analyses of metastatic lymph nodes and primary tumors, we prioritized SPIB, an ETS-family transcription factor previously implicated in immune cell differentiation^44^^,^^45^ and context-dependent oncogenic roles in solid tumors,^46^^,^^47^^,^^48^^,^^49^ as a candidate regulator of lymphatic dissemination. Furthermore, Regulon activity analysis (SCENIC) confirmed SPIB’s transcriptional activation in metastatic epithelial cells, and IHC validation in our GC patient cohort substantiated its elevated protein expression specifically in lymph node metastases. Together, these findings highlight SPIB as a promising regulatory candidate in metastatic remodeling, offering a valuable foundation for future studies to further explore its functional and clinical significance in GC.

In summary, we developed an ensemble-based, 11-gene LNM-related prognostic signature that robustly predicts survival and offers significant clinical utility for patient stratification across eight independent cohorts. SPIB was then identified as a potential candidate regulator of epithelial plasticity and lymphatic dissemination, supported by regulon inference and corroborative IHC validation. Collectively, our study offers both a robust prognostic tool and mechanistic insights into the molecular landscape of lymphatic dissemination, nominating SPIB as a candidate for future functional validation in the context of metastatic progression in GC.

### Limitations of the study

Several limitations should be acknowledged. First, although the LMGS model demonstrated robust prognostic performance across multiple independent cohorts, it exhibited limited statistical significance in fixed-threshold KM analysis within the KUCM and MDACC cohorts. This discrepancy may stem from differences in patient characteristics, TNM stage distribution, and treatment eras across various centers. Moreover, the restricted sample sizes in these specific cohorts potentially reduced the statistical power for categorical risk stratification. Notably, our continuous variable modeling confirmed that the LMGS remains a robust prognostic indicator across nearly all datasets, suggesting that the lack of significance in certain KM analyses may be partially explained by dichotomization-induced information loss. The drug sensitivity and immunotherapy response analyses were based on in silico estimations. While these results provide valuable insights, they are primarily hypothesis-generating and require further prospective clinical validation. Second, while SPIB was identified as a potential regulator of lymphatic dissemination, its mechanistic role in GC metastasis remains to be functionally validated, particularly through *in vivo* models and patient-derived organoids. Third, the scRNA-seq and spatial transcriptomics analyses were performed on limited samples, which may not fully capture the inter-patient heterogeneity or immune contexture. The observed associations between LMGS activity and immune cell subsets at the tumor-normal interface are correlative; direct causal relationships and specific interactions between LMGS-related genes and immune cells require further experimental validation. Future studies should prioritize functional experiments to elucidate the mechanistic roles of SPIB and LMGS-related genes in immune modulation, epithelial plasticity, and metastatic seeding.

## Resource availability

### Lead contact

Further information and requests for resources should be directed to and will be fulfilled by the lead contact, Yanfeng Hu (banby@smu.edu.cn).

### Materials availability

This study did not generate any new materials.

### Data and code availability

Public datasets analyzed in this study include The Cancer Genome Atlas Stomach Adenocarcinoma (TCGA-STAD), the Genotype-Tissue Expression (GTEx: https://xenabrowser.net/datapages/), and multiple Gene Expression Omnibus (GEO) cohorts (GSE62254, GSE26899, GSE13861, GSE26901, GSE26253, GSE28541, GSE84437, GSE163558, and GSE251950). The in-house transcriptomic data of paired primary GC and metastatic lymph node samples are publicly available from the GEO under the accession no. GSE246963. To ensure absolute transparency and computational reproducibility, a comprehensive, step-by-step Reproducibility Protocol and Analytical Pipeline is provided as a standalone file (Document S2). Furthermore, the modular, fully executable R scripts required to calculate the LMGS risk scores and reproduce our primary stratification results have been deposited in a public GitHub repository (https://github.com/doggyzz001/LMGS-Gastric-Cancer-Pipeline). Further details are provided in the key resources table, and any additional information required to reanalyze the data reported in this paper is available from the lead contact upon request.

## Acknowledgments

The authors gratefully acknowledge the institutional support from the 10.13039/501100010112Nanfang Hospital and the resources provided for this study. This study received financial support from the 10.13039/100014718National Natural Science Foundation of China (82272062), Development Project of the 10.13039/100021065Gastrointestinal Oncology Research Institute of Nanfang Hospital (J1010171102), The 2025 National Innovation and Entrepreneurship Training Program for Undergraduates at the 10.13039/501100010096Southern Medical University (202512121009) and Special Funds for the Cultivation of 10.13039/501100013283Guangdong College Students Scientific and Technological Innovation (pdjh2025bj051).

## Author contributions

Conceptualization, Z.D. and K.H.; methodology, Z.D., K.H., and D.C.; formal analysis, Z.D., K.H., and D.C.; investigation, L.L., M.C., S.Z., and J.Z.; resources, L.L. and Y.Z.; writing – original draft, Z.D. and K.H.; writing – review and editing, K.H., D.C., and G.S.; visualization, Z.D. and K.H.; supervision, G.S. and Y.Z.; funding acquisition, Y.H. and G.S. All authors read and approved the final manuscript.

## Declaration of interests

The authors declare no competing interests.

## STAR★Methods

### Key resources table

**Table:** 

REAGENT or RESOURCE	SOURCE	IDENTIFIER
**Antibodies**		

SPIB Polyclonal antibody	proteintech	15768-1-AP
Universal two-step IHC detection kit	ZSGB-BIO	PV-9000
DAB detection kit	ZSGB-BIO	ZLI-9018

**Biological samples**		

Formalin-fixed paraffin-embedded gastric cancer and matched MLN sections	Department of Pathology, Nanfang hospital, Southern Medical University	N/A

**Deposited data**		

TCGA-STAD cohort database	The Cancer Genome Atlas Program	https://tcga-data.nci.nih.gov/tcga/
ACRG cohort database	Gene Expression Omnibus	https://www.ncbi.nlm.nih.gov/geo/query/acc.cgi?acc=GSE62254
KUGH cohort database	Gene Expression Omnibus	https://www.ncbi.nlm.nih.gov/geo/query/acc.cgi?acc=GSE26899
YUHS cohort database	Gene Expression Omnibus	https://www.ncbi.nlm.nih.gov/geo/query/acc.cgi?acc=GSE13861
KUCM cohort database	Gene Expression Omnibus	https://www.ncbi.nlm.nih.gov/geo/query/acc.cgi?acc=GSE26901
SMC cohort database	Gene Expression Omnibus	https://www.ncbi.nlm.nih.gov/geo/query/acc.cgi?acc=GSE26253
MDACC cohort database	Gene Expression Omnibus	https://www.ncbi.nlm.nih.gov/geo/query/acc.cgi?acc=GSE28541
GSE84437 cohort database	Gene Expression Omnibus	https://www.ncbi.nlm.nih.gov/geo/query/acc.cgi?acc=GSE84437
GTEx database	UCSC Xena browser	https://xenabrowser.net/datapages/
Raw data – scRNA-seq (Human gastric cancer samples with matched metastatic lymph nodes)	Gene Expression Omnibus	https://www.ncbi.nlm.nih.gov/geo/query/acc.cgi?acc=GSE163558
Raw data – spatial transcriptomics (Human gastric cancer samples)	Gene Expression Omnibus	https://www.ncbi.nlm.nih.gov/geo/query/acc.cgi?acc=GSE251950
Raw data – bulkRNA-seq (Human gastric cancer samples with matched metastatic lymph nodes)	Gene Expression Omnibus	https://www.ncbi.nlm.nih.gov/geo/query/acc.cgi?acc=GSE246963

**Software and algorithms**		

R (4.3.3)	The R Foundation	https://www.r-project.org/
Python 3.11	Python	https://www.python.org/
limma R package(3.58.1)	Ritchie et al.^50^	https://bioconductor.org/packages//release/bioc/html/limma.html
EnhancedVolcano R package(1.22.0)	Blighe et al.^51^	https://github.com/kevinblighe/EnhancedVolcano
pheatmap R package(1.0.13)	Kolde et al.^52^	https://github.com/raivokolde/pheatmap
VennDiagram R package(1.7.3)	Chen et al.^53^	https://github.com/uclahs-cds/package-VennDiagram
WGCNA R package(1.73)	Langfelder et al.^54^	https://cran.r-project.org/web/packages/WGCNA/
randomForestSRC R package(3.4.4)	Ishwaran et al.^55^	https://www.randomforestsrc.org/index.html
glmnet R package(4.1–10)	Friedman et al.^56^	https://github.com/cran/glmnet
ggDCA R package(1.2)	Zhang et al.^57^	https://github.com/yikeshu0611/ggDCA
rms R package(6.8.1)	Harrell et al.^58^	https://cran.r-project.org/web/packages/rms/index.html
survival R package(3.8–3)	Therneau et al.^59^	https://cran.r-project.org/web/packages/survival/index.html
timeROC R package(0.4)	Blanche et al.^60^	https://cran.r-project.org/web/packages/timeROC/index.html
IOBR R package(0.99.0)	Zeng et al.^61^^,^^62^	https://github.com/IOBR/IOBR
oncoPredict R package(1.2)	Maeser et al.^63^	https://github.com/HuangLabUMN/oncoPredict
maftools R package(2.20.1)	Mayakonda et al.^64^	https://bioconductor.org/packages/release/bioc/html/maftools.html
Seurat R package(5.3.1)	Hao et al.^65^	https://satijalab.org/seurat/
Harmony R package(1.2.4)	Korsunsky et al.^66^	https://portals.broadinstitute.org/harmony/
SingleR R package(2.4.1)	Aran et al.^67^	https://bioconductor.org/packages//release/bioc/html/SingleR.html
scRNAtoolVis R package(0.1.0)	Zhang J^68^	https://junjunlab.github.io/scRNAtoolVis-manual/index.html
InferCNV R package(1.18.1)	Tickle et al.^69^	https://github.com/broadinstitute/inferCNV
CytoTRACE2 R package(1.0.0)	Kang et al.^70^	https://github.com/digitalcytometry/cytotrace2
Monocle 2 R package(2.30.1)	Qiu et al.^71^	https://cole-trapnell-lab.github.io/monocle-release/
CellChat R package(1.6.1)	Jin et al.^72^	https://github.com/jinworks/CellChat
pySCENIC(0.12.1)	Van et al.^73^	https://github.com/aertslab/pySCENIC
ggplot2 R package(3.4.2)	Wickham^74^	https://cran.r-project.org/web/packages/ggplot2/index.html
ggridges R package(0.5.7)	Wilke et al.^75^	https://wilkelab.org/ggridges/
Scanpy(1.10.0)	Wolf et al.^76^	https://scanpy.readthedocs.io/en/stable/
Hotspot(0.9.0)	DeTomaso et al.^77^	https://hotspot.readthedocs.io/en/latest/

### Experimental model and study participant details

#### Human specimens

A total of 20 sets of formalin-fixed paraffin-embedded (FFPE) sections, including primary GC tissues and matched metastatic lymph nodes, were obtained from the Department of Pathology at Nanfang Hospital for immunohistochemistry (IHC) staining. All procedures involving human participants were conducted in accordance with national regulations and the principles of the Declaration of Helsinki. Ethical approval was granted by the Nanfang Medical Ethics Committee (Approval No.: NFEC-2025-498). Written informed consent was obtained from all participants prior to sample collection.

### Method details

#### Data collection and preprocessing

Transcriptomic data from multiple publicly available GC cohorts were utilized in this study. Specifically, we included RNA-seq data from The Cancer Genome Atlas Stomach Adenocarcinoma (TCGA-STAD) and seven Gene Expression Omnibus (GEO) datasets: KUGH (GSE26899), YUHS (GSE13861), KUCM (GSE26901), SMC (GSE26253), MDACC (GSE28541), ACRG (GSE62254), and GSE84437. Additionally, RNA-seq data from eight paired primary GC and matched metastatic lymph node samples previously generated by our group (GEO accession: GSE246963) were incorporated. Normal stomach tissue RNA-seq data from the Genotype-Tissue Expression (GTEx) project were retrieved via the UCSC Xena browser(https://xenabrowser.net/datapages/). Somatic mutation data were also obtained from TCGA.

All raw RNA-seq count data from TCGA-STAD and GTEx stomach were normalized to transcripts per million (TPM) and log_2_-transformed. Affymetrix microarray expression data from the GEO cohorts were preprocessed using the limma R package,^50^ including background correction, quantile normalization, and probe summarization. To ensure data consistency, we performed normalization within each individual dataset using the normalizeBetweenArrays function from the limma package, followed by log_2_ transformation. Finally, we separately processed single-cell RNA-seq data from three primary gastric tumors with two matched metastatic lymph node samples (GEO accession: GSE163558), as well as spatial transcriptomics data from nine GC patients (GEO accession: GSE251950), following the procedures detailed below. Samples with missing clinical outcomes or incomplete transcriptomic profiles were excluded during the initial data preprocessing phase.

#### Differential expression analysis of primary tumor and metastatic lymph node samples

Differential expression analysis was conducted using the limma package in R.^50^ Genes with low expression levels were filtered out prior to analysis. The data were normalized and transformed using the voom method to account for mean–variance relationships and heteroscedasticity. Linear modeling was performed with lmFit, followed by contrast fitting to identify genes significantly differentially expressed between primary tumor and metastatic lymph node samples. Genes exhibiting an absolute log_2_ fold change greater than 1 and an adjusted *p*-value (Benjamini–Hochberg corrected) less than 0.05 were defined as differentially expressed (Table S1). Visualization of these results was performed using the EnhancedVolcano,^51^ pheatmap,^52^ and VennDiagram^53^ R packages.

#### Weighted correlation Network Analysis (WGCNA)

Coexpression networks were constructed using mRNA expression data from the ACRG dataset with the WGCNA package in R.^54^ An appropriate soft-thresholding power (β) was selected to approximate scale-free topology. The weighted adjacency matrix was subsequently transformed into a topological overlap matrix (TOM), from which the corresponding dissimilarity measure (1 – TOM) was derived. Gene modules were identified via dynamic tree cutting. The module exhibiting the strongest correlation with lymphatic metastasis was selected for further investigation. Within this module, genes demonstrating both high gene significance (GS) and high module membership (MM) were classified as lymph metastasis–related genes (Table S2).

#### Functional enrichment analysis

Following the identification of lymph metastasis–related genes through intersection and univariate Cox regression analysis, functional enrichment analyses were conducted to elucidate their biological roles. Gene Ontology (GO) and Kyoto Encyclopedia of Genes and Genomes (KEGG) pathway enrichment analyses were performed using the DAVID Bioinformatics Resources (https://davidbioinformatics.nih.gov/).

#### Prognostic signature generation and validation

To enhance predictive accuracy and model robustness, we implemented a comprehensive machine learning framework by integrating 10 algorithms into 101 combinations^26^ to develop a prognostic signature for GC. These included random survival forest (RSF),^55^ elastic net (Enet),^56^ Lasso,^56^ Ridge regression,^78^ stepwise Cox regression,^79^ CoxBoost,^80^ partial least-squares regression for Cox (plsRcox),^81^ supervised principal components (SuperPC),^82^^,^^83^ generalized boosted regression modeling (GBM),^84^ and survival support vector machine (survival-SVM).^85^^,^^86^^,^^87^

The signature was generated through the following pipeline: (a) prognostic genes were identified via univariate Cox regression in the ACRG (GSE62254) dataset (Table S3); (b) all algorithm combinations were trained using leave-one-out cross-validation (LOOCV); (c) performance was evaluated across eight datasets, including the training cohort and seven external validation sets (TCGA-STAD, KUGH, YUHS, KUCM, SMC, MDACC, and GSE84437); (d) Harrell’s concordance index (C-index) was used to assess performance, and the model with the highest average C-index was selected as the final model. Based on the highest average C-index, the ensemble of RSF and ENet was selected as the final model. For the specific implementation of this ensemble, RSF was configured with 1,000 trees and a minimum node size of 10 to identify the 34 most informative prognostic variables. These features were subsequently refined using ENet regression (alpha = 0.5), where the best-fit regularization parameter lambda was identified via internal 10-fold cross-validation using the lambda.min criterion. Crucially, each validation cohort remained entirely independent; no data merging or cross-cohort batch correction was performed, thereby strictly testing the cross-platform generalizability of the LMGS.

#### Clinical utility and robustness assessment

Clinical net benefit of the LMGS was quantified via decision curve analysis (DCA) using the ggDCA R package,^57^ with comparisons made between the risk score, AJCC TNM staging, and an integrated model across 1-, 3-, and 5-year overall survival (OS) intervals. Model calibration was assessed using the rms R package^58^ to compare predicted and observed survival probabilities, employing 1,00 bootstrap resamples for internal validation across all cohorts. For robustness testing, the LMGS score was treated as a continuous variable in univariate Cox proportional hazards regression using the survival R package.^59^ Hazard ratios (HR) and 95% confidence intervals (CI) were calculated for each cohort and visualized via forest plots to evaluate prognostic stability across independent datasets.

#### Immune infiltration analysis and drug sensitivity prediction

To characterize the tumor immune microenvironment, we used the IOBR R package,^61^^,^^62^ which integrates multiple deconvolution algorithms, including MCP-counter,^88^ EPIC,^89^ xCell,^90^ CIBERSORT,^91^ IPS,^92^ quanTIseq,^93^ ESTIMATE,^94^ and TIMER.^95^ This allowed us to systematically estimate immune cell composition and immune-related signatures across GC cohorts. To predict therapeutic response, we employed the oncoPredict R package,^63^ which utilizes gene expression data and the Genomics of Drug Sensitivity in Cancer (GDSC) database to estimate half-maximal inhibitory concentration (IC50) values across a broad spectrum of anticancer agents.

#### Somatic mutation analysis

Somatic mutation data from the TCGA-STAD cohort were analyzed using the maftools R package.^64^ Key metrics, including mutational frequency, tumor mutational burden (TMB), and significantly mutated genes, were calculated and visualized to characterize the genomic landscape of GC.

#### Single-cell RNA sequencing data processing and quality control

Single-cell RNA sequencing (scRNA-seq) data were processed using the Seurat v5 R package.^65^ Raw count matrices were imported via CreateSeuratObject, retaining genes detected in ≥3 cells and cells expressing ≥300 genes. Low-quality cells were removed based on the following criteria: <300 or >7,000 detected genes, >10% mitochondrial gene content (mt%), >3% hemoglobin gene content (HB%), total RNA counts (nCount_RNA) < 1,000 or above the 97th percentile (to remove low-quality cells and potential doublets).To correct for batch effects, individual Seurat objects were merged and harmonized using the Harmony algorithm.^66^ The integrated dataset was then normalized and scaled for downstream analysis.

#### Unsupervised clustering and dimensionality reduction

After integration, the dataset was normalized (NormalizeData) and 3,000 highly variable genes were selected using the FindVariableFeatures function with the “vst” method. Data were scaled using ScaleData, and dimensionality reduction was performed via principal component analysis (PCA), retaining the top 40 principal components (PCs). A K-nearest neighbors (KNN) graph was constructed (FindNeighbors), followed by graph-based clustering (FindClusters) at a resolution of 0.2. Dimensionality reduction and visualization were performed using UMAP via the RunUMAP function, with PCs consistent with those used for clustering.

Initial cell-type annotation was performed using SingleR R package,^67^ followed by refinement based on top marker genes identified by FindAllMarkers. Annotation accuracy was further ensured through a systematic manual review of established GC literature and cross-validated using canonical cell-type marker references and curated databases (e.g., CellMarker2.0 and PanglaoDB). To ensure quality, cells with elevated mitochondrial content (≥10%) were excluded post-clustering. The filtered dataset was then re-clustered using the same pipeline for final visualization and differential expression analysis. All scRNA-seq figures were visualized and refined using the scRNAtoolVis package61.

#### Identification of marker genes

To characterize each cell cluster, differentially expressed marker genes were identified using the FindAllMarkers function in Seurat. Marker selection was based on differences in average expression levels (log fold change), with statistical significance evaluated using the Wilcoxon rank-sum test. Default parameters were applied: min.pct = 0.25 and logfc.threshold = 1.0.

#### Re-clustering of epithelial cells and identification of malignant subpopulations

Epithelial cells were subset from the integrated Seurat object and re-analyzed following the same preprocessing pipeline as in the initial clustering. After normalization and scaling via ScaleData, dimensionality reduction was performed using PCA, and unsupervised clustering was conducted with FindClusters.

We conducted single-cell copy number variation (CNV) analysis using the InferCNV R package.^69^ Non-epithelial cells served as the reference population. CNV scores were computed for each epithelial cell based on InferCNV’s output, and cluster-level CNV burden was summarized accordingly. Clusters exhibiting markedly elevated CNV scores, which indicate chromosomal instability, were classified as malignant epithelial subpopulations63.

#### Cell developmental trajectory

To explore the developmental dynamics of epithelial cells, we first assessed cell differentiation potential using CytoTRACE2,^70^ which enabled the inference of putative progenitor-like states based on transcriptional diversity. Subsequently, Monocle 2^71^ was employed to construct a pseudotime trajectory of epithelial subpopulations. The integrated Seurat object was converted into a Monocle 2-compatible CellDataSet. Trajectory inference was conducted using highly variable genes, and the root state was defined based on CytoTRACE2-inferred differentiation potential, representing the earliest developmental state for pseudotime ordering.

#### Cell–cell interaction analysis

Cell–cell communication within metastatic lymph nodes was analyzed using the CellChat R package (v1.6.1),^72^ leveraging a curated ligand–receptor interaction database (CellChatDB.human) to infer intercellular signaling networks based on known molecular interactions. To minimize noise, interactions involving fewer than 10 cells were excluded using the filterCommunication function. Communication probabilities were computed at the signaling pathway level using computeCommunProbPathway, and networks were aggregated across cell types with the aggregateNet function. Centrality metrics were then calculated via netAnalysis_computeCentrality, allowing for the identification of key signaling pathways and dominant sender–receiver interactions among cell populations.

#### Differential expression of epithelial cells

Differentially expressed genes (DEGs) among epithelial cell subpopulations were identified using the FindMarkers and FindAllMarkers functions in Seurat. Genes with a log fold change threshold (logfc.threshold) of ≥0.25 were considered significant (Table S4).

#### Transcription factor regulatory analysis

The raw count matrix of epithelial cells was extracted from the Seurat object in R using the LayerData function. The matrix was then exported to Python and converted into a.loom file format using the loompy library (https://github.com/linnarsson-lab/loompy). The resulting loom file was processed through the pySCENIC pipeline^73^ to infer transcription factor (TF) activity and gene regulatory networks. Visualization of TF activity was performed in R using the ggplot2,^74^ pheatmap,^52^ and ggridges^75^ packages.

#### Spatial transcriptomics data processing

Spatial transcriptomic data were processed using Scanpy^76^ in Python. Low-quality spots and genes were excluded by filtering out genes detected in fewer than 10 spots and spots expressing fewer than 500 genes. Spots with >50% mitochondrial transcript proportion were removed to eliminate low-quality capture regions. Gene expression counts were normalized to 10,000 transcripts per spot, log-transformed, and scaled after regressing out total counts and mitochondrial percentage. Highly variable genes were selected (mean expression between 0.0125 and 3, dispersion >0.5) for dimensionality reduction by principal component analysis (PCA). Batch correction across spatial samples was performed using Harmony integration. A neighborhood graph was then constructed, and spatial domains were identified using the Leiden clustering algorithm (resolution = 0.4), defining distinct molecular niches.

#### Identification of spatial transcriptomics hotspot

Spatial gene module scores were computed using the Hotspot^77^ framework, which identifies spatially autocorrelated gene expression patterns. A k-nearest neighbor graph (k = 30) was constructed based on spatial embeddings, and neighborhood weights were modeled using a distance-aware negative binomial (danb) kernel with a neighborhood factor of 6. Gene-wise autocorrelation scores were subsequently derived from the fitted model.

#### Immunohistochemistry

FFPE sections were deparaffinized, rehydrated, and underwent heat-induced antigen retrieval in 10 mM citrate buffer (pH 6.0) for 15–20 min. After blocking endogenous peroxidase with 3% H_2_O_2_ and non-specific binding with 5% normal goat serum, slides were incubated with primary antibodies against SPIB (dilution [1:200]) overnight at 4°C. Immunodetection was performed using a horseradish peroxidase (HRP)-conjugated polymer system according to the manufacturer’s instructions. Signals were visualized with DAB and counterstained with hematoxylin.

### Quantification and statistical analysis

#### IHC scoring and evaluation

Immunohistochemistry (IHC) evaluation was performed using a semi-quantitative scoring system based on a combination of staining intensity and the percentage of positive cells. Staining intensity was graded as follows: 0 (no staining/negative), 1 (light yellow/weakly positive), 2 (brownish-yellow/moderately positive), and 3 (dark brown/strongly positive). The percentage of positive cells was scored as 1 (1%–25%), 2 (26%–50%), 3 (51%–75%), and 4 (76%–100%). The final immunoreactivity score (IHC score) was calculated by multiplying the staining intensity score by the score for the percentage of positive cells.

#### Statistical analysis

All statistical analyses and visualizations were performed in R (version 4.3.3). Categorical variables were compared using the chi-squared test, while continuous variables were analyzed using either the Wilcoxon rank-sum test or the Student’s *t* test, as appropriate. Cox proportional hazards regression and Kaplan–Meier survival analysis were performed using the survival package.^59^ Time-dependent receiver operating characteristic (ROC) curve analyses were conducted using the timeROC package^60^ to evaluate prognostic accuracy. All statistical tests were two-sided, with a significance threshold set at *p* < 0.05. Additionally, the Benjamini-Hochberg (BH) method was utilized for multiple testing corrections in high-dimensional analyses to control the false discovery rate. A *p*-value or adjusted *p*-value <0.05 was considered statistically significant throughout the study(∗*p* < 0.05; ∗∗*p* < 0.01; ∗∗∗*p* < 0.001; and NS *p* > 0.05).

## Supplemental information

Document S1. Figures S1–S4

Table S1. Significantly differentially expressed genes between paired primary gastric tumors and metastatic lymph nodes in our cohort, related to Figure 1

Table S2. WGCNA-identified genes associated with lymph node metastasis in the ACRG cohort, related to Figure 1

Table S3. Univariate Cox regression analysis of lymph metastasis-related genes in the ACRG cohort, related to Figure 2

Table S4. Significantly up-regulated genes in the LN-Epi_2, related to Figure 8
