# Supplementary material for: Multiomics identifies a prognostic signature and SPIB as a potential regulator of gastric cancer lymph node metastasis
Source: iScience. 2026 Jul 16;29(8):116774. doi: 10.1016/j.isci.2026.116774 (PMC13383869; doi:10.1016/j.isci.2026.116774)
Supplement: Document S1. Figures S1–S4 [file mmc1.pdf]

**iScience, Volume *29***

## Supplemental information

# Multiomics identifies a prognostic signature and SPIB as a potential regulator of gastric cancer lymph node metastasis

**Zhijie Duan, Keyu He, Dianjie Chen, Lifan Lin, Ming Cao, Shaowei Zhang, Junbo Zhuang, Yintong Zhu, Guodong Shen, and Yanfeng Hu**

# Supporting Information

- **Figure S1**
- **Figure S2**
- **Figure S3**
- **Figure S4**
- **Full Reproducibility Pipeline**

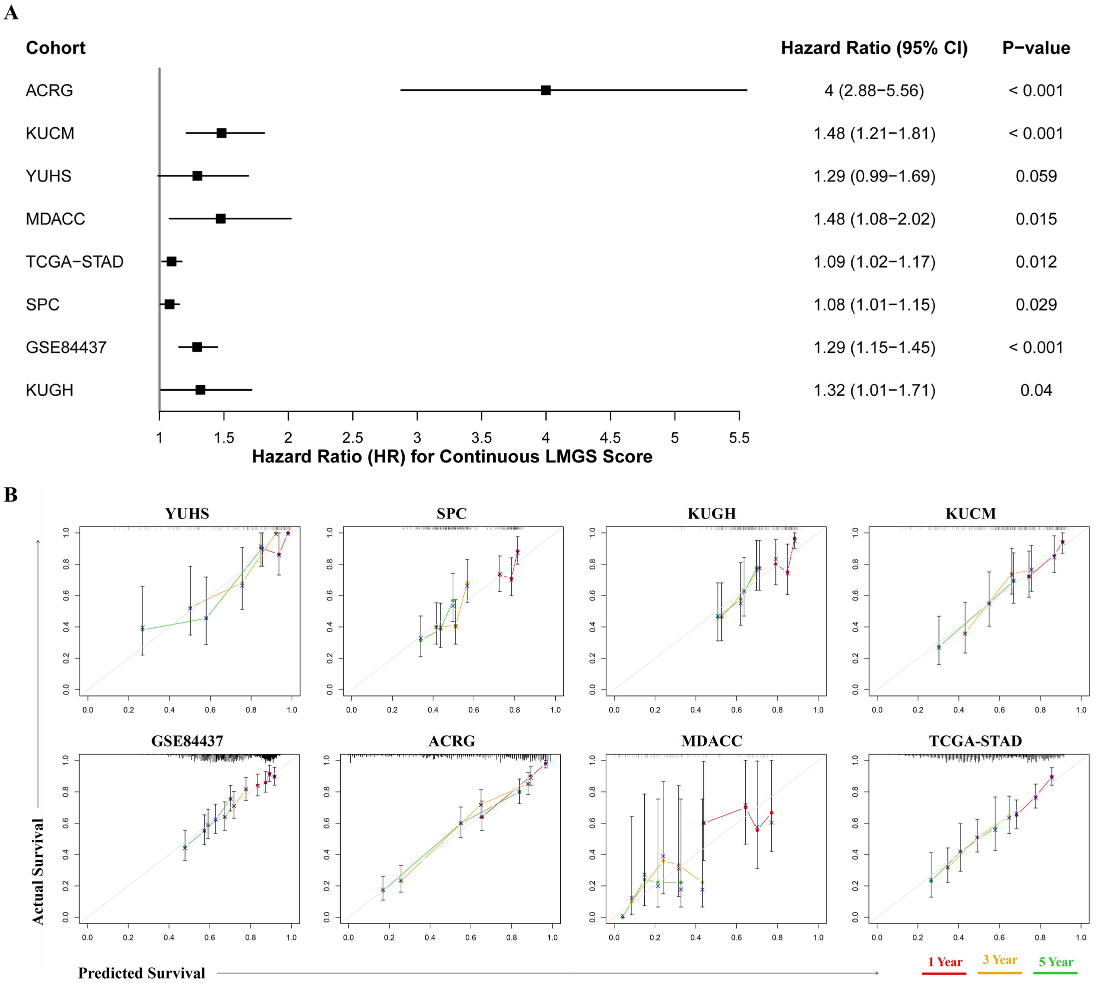


**Figure S1. Multi-dimensional validation and clinical utility of the LMGS, Related to Figure 2**
**A.** Forest plot of univariate Cox regression analysis evaluating the LMGS score as a continuous predictor across all cohorts. **B.** Calibration plots comparing the predicted and actual overall survival (OS) probabilities at 1, 3, and 5 years across all cohorts.

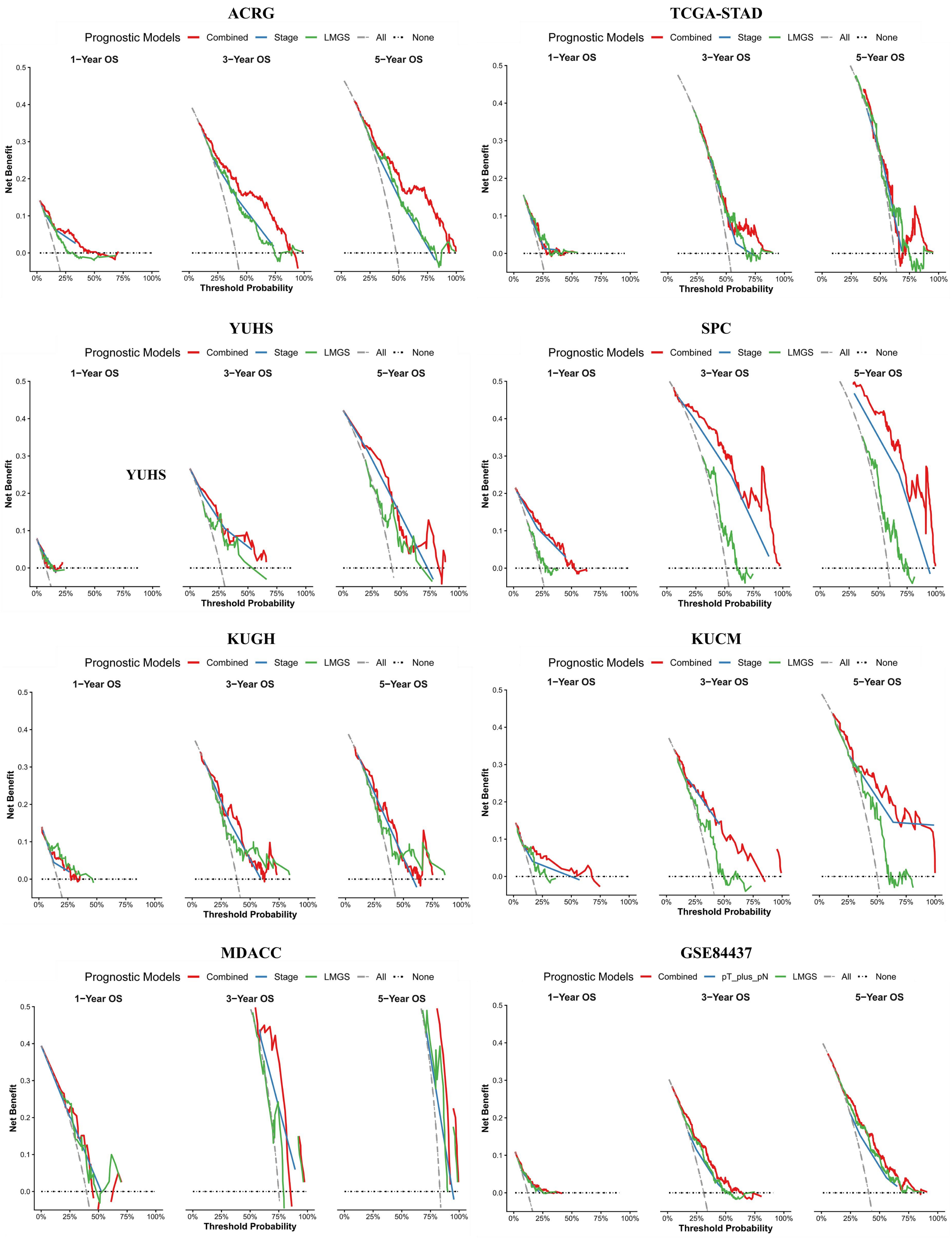


**Figure S2. Decision Curve Analysis (DCA) of the LMGS across multi-center cohorts, Related to Figure 2**

DCA curves are presented for the training and seven validation cohorts (labeled accordingly) at 1-year, 3-year, and 5-year overall survival (OS) time points. The red line represents the integrated model (LMGS plus clinical parameters), the green line represents the LMGS alone, and the blue line represents clinical parameters alone (AJCC stage, or pT plus pN for the GSE84437 cohort). The gray and black lines represent the "Treat All" and "Treat None" strategies, respectively. The y-axis indicates the net benefit, and the x-axis indicates the threshold probability.

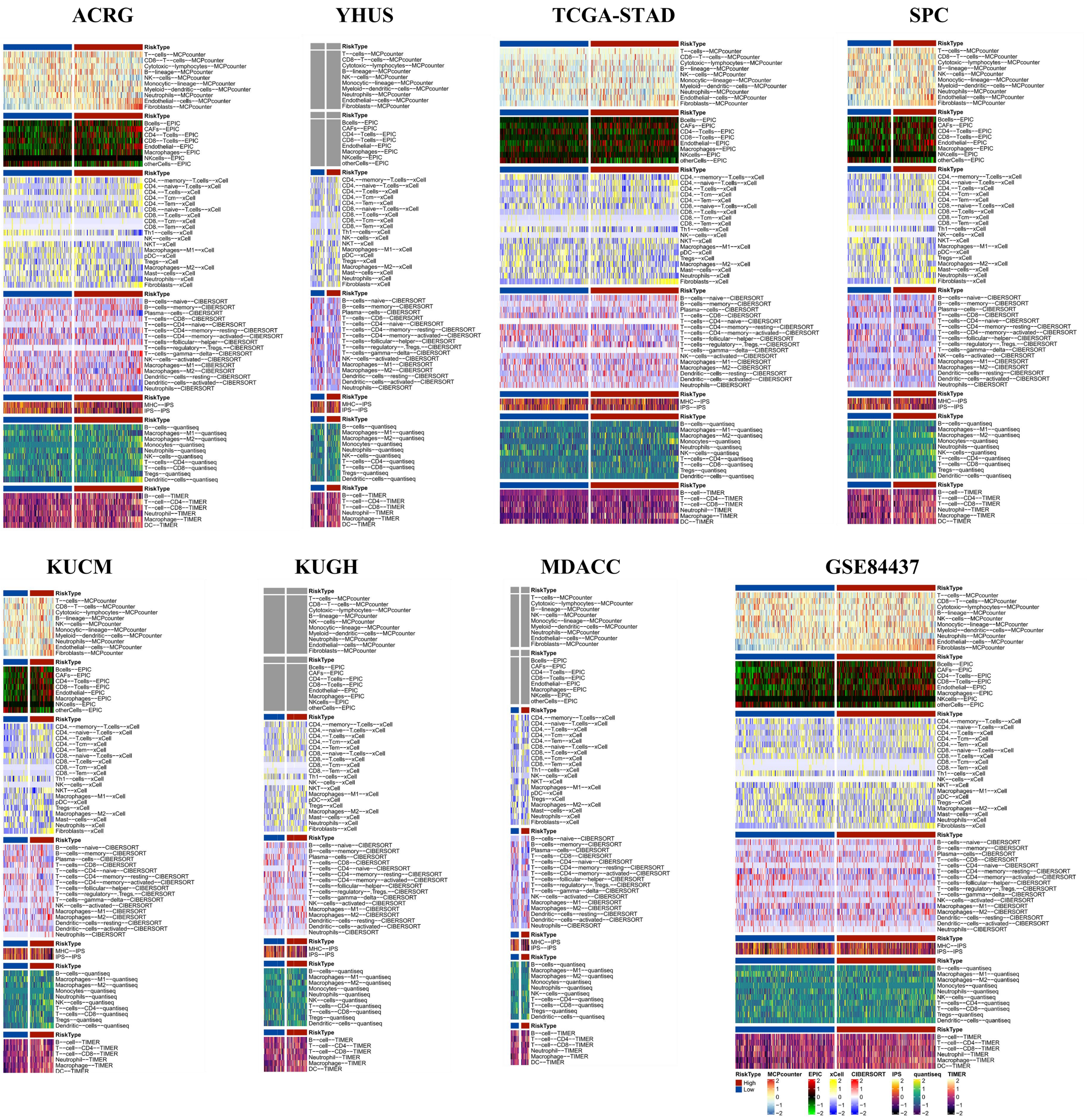


**Figure S3. Immune infiltration levels in high and low LMRS groups across multiple cohorts, Related to Figure 3**

Immune cell abundances were assessed using multiple algorithms (mcpcounter, xcell, cibersort, etc.) to compare immune infiltration between high and low LMRS groups (Gray shading indicates cell types that were not successfully deconvoluted by the algorithm).

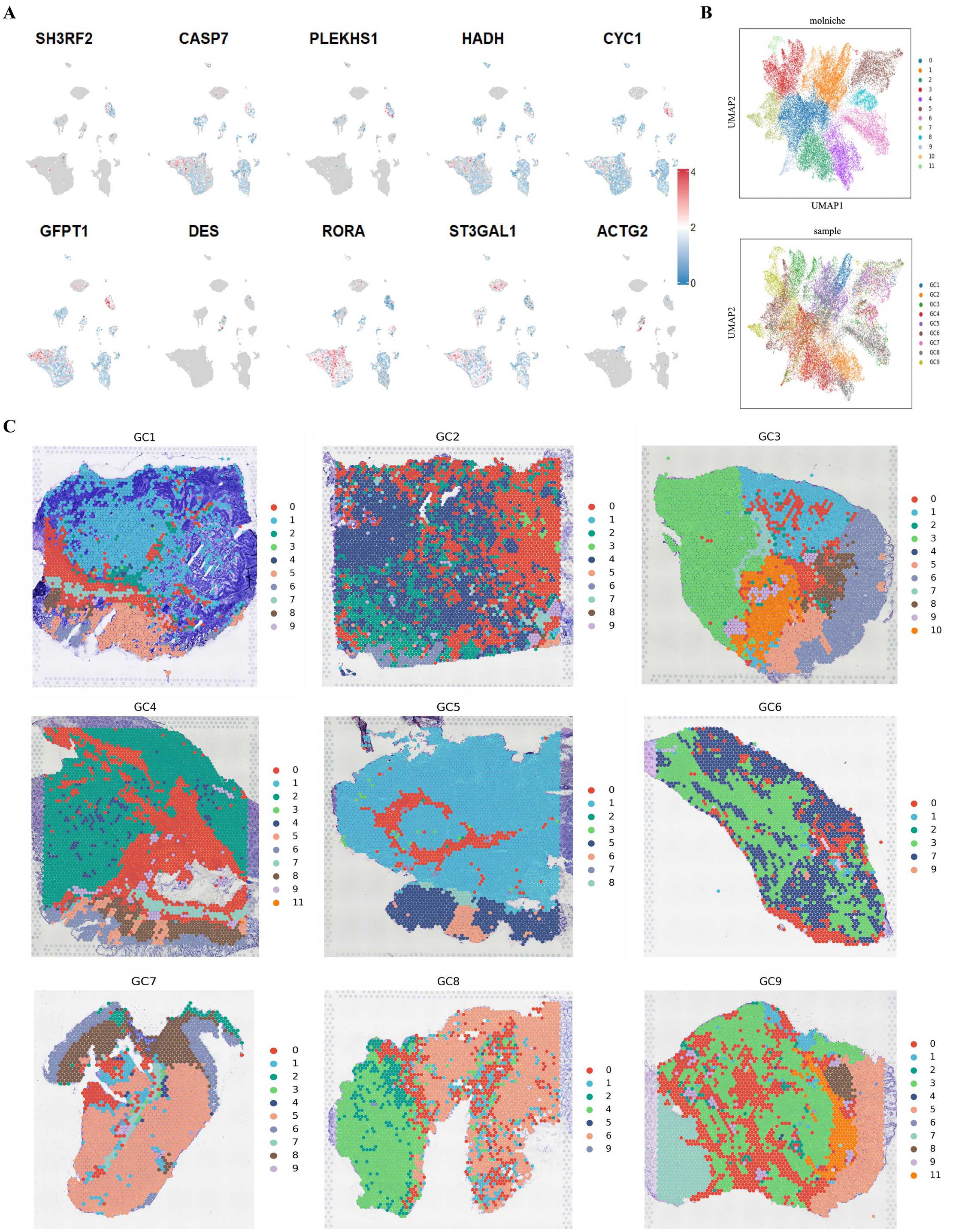


**Figure S4. Single-cell expression profiles of LMGS genes and identification of spatial molecular niches in GC, Related to Figure 6**

**A.** Feature plots of LMGS gene expression across single-cell populations. **B.** UMAP visualization of spatial molecular niche classification across samples. **C.** Spatial localization of molecular niches in representative tissue sections.

**Full Reproducibility Pipeline**

**Section 0: Overview and File Structure**

To enable independent verification of our primary findings, this note provides a fully self-contained, step-by-step computational pipeline. The workflow is organized into seven sections: (0) file structure overview, (1) software environment, (2) input data format, (3) data preprocessing, (4) candidate gene identification, (5) LMGS construction and risk score calculation, and (6) model evaluation and key figure reproduction. Each section builds sequentially on the previous one.

**Expected project directory layout:**

```
project_LMGS/
├── data/
│   ├── raw/
│   │   ├── data_counts.txt        # in-house paired RNA-seq count matrix (GSE246963)
│   │   ├── tumor_metadata.txt    # sample IDs for primary tumor group (8 samples)
│   │   ├── mln_metadata.txt       # sample IDs for metastatic lymph node group (8 samples)
│   │   ├── ACRG_GSE62254_expression.csv # genes × samples expression matrix
│   │   ├── ACRG_GSE62254_clinical.csv      # OS.time (days), OS (0/1), Stage, pT, pN, etc
│   │   ├── TCGA_STAD_expression.csv
│   │   ├── TCGA_STAD_clinical.csv
│   │   ├── KUCM_GSE26901_expression.csv
│   │   ├── KUCM_GSE26901_clinical.csv
│   │   ├── YUHS_GSE13861_expression.csv
│   │   ├── YUHS_GSE13861_clinical.csv
│   │   ├── MDACC_GSE28541_expression.csv
│   │   ├── MDACC_GSE28541_clinical.csv
│   │   ├── SPC_GSE15459_expression.csv
│   │   ├── SPC_GSE15459_clinical.csv
│   │   ├── GSE84437_expression.csv
│   │   ├── GSE84437_clinical.csv # staging uses pT + pN instead of composite Stage
│   │   ├── KUGH_GSE26899_expression.csv
│   │   └── KUGH_GSE26899_clinical.csv
│   └── processed/
│       └── [cohort]_clean.rds                          # output of Section 3
├── output/
│   ├── DEG_results.txt                                  # output of Section 4.1
│   ├── WGCNA_modules.rds                                # output of Section 4.2
│   ├── prognostic_91genes.csv                           # output of Section 4.4
│   ├── LMGS_11genes_coefficients.csv                    # output of Section 5.2
│   ├── risk_scores_all_cohorts.csv                      # output of Section 5.3
│   └── figures/
│       ├── Figure_KM_[cohort].pdf                       # output of Section 6.1
│       ├── Figure_Calibration_[cohort].pdf              # output of Section 6.3
│       ├──Figure_DCA_[cohort].pdf                       # output of Section 6.4
```

**Section 1: Software Environment**

All analyses were performed in R (version 4.3.3). Two global random seeds were used throughout: set.seed(321) for all machine learning steps and set.seed(54321) for WGCNA.

| Package | Version | Source | Purpose |
|---|---|---|---|
| R | 4.3.3 | https://cran.r-project.org | Base environment |
| limma | 3.58.1 | Bioconductor | Normalization, DEA |
| edgeR | 4.0.16 | Bioconductor | Count filtering, normalization |
| WGCNA | 1.73 | CRAN | Co-expression network analysis |
| glmnet | 4.1-10 | CRAN | Elastic Net regression |
| randomForestSRC | 3.4.4 | CRAN | Random Survival Forest |
| survivalsvm | 0.0.6 | CRAN | Survival SVM |
| superpc | 1.12 | CRAN | Supervised PCA |
| plsRcox | 1.8.0 | CRAN | PLS Cox regression |
| gbm | 2.2.2 | CRAN | Gradient Boosting Machine |
| survival | 3.8-3 | CRAN | Survival modeling |
| timeROC | 0.4 | CRAN | Time-dependent AUC |
| rms | 6.8.1 | CRAN | Calibration curves |
| ggDCA | 1.2 | CRAN | Decision Curve Analysis |
| survminer | 0.4.9 | CRAN | KM plot visualization |

Installation commands:

```
#R
install.packages(c("WGCNA", "glmnet", "randomForestSRC", "CoxBoost",
                   "survivalsvm", "superpc", "plsRcox", "gbm",
                   "survival", "timeROC", "rms", "ggDCA", "survminer"))
if (!requireNamespace("BiocManager")) install.packages("BiocManager")
BiocManager::install(c("limma", "edgeR"))
```

**Section 2: Input Data Format Specification**

Expression matrices must have gene symbols as row names and sample IDs as column names:

| | Sample_1 | Sample_2 | Sample_3 |
|---|---|---|---|
| GENE_A | x.xxx | x.xxx | x.xxx |
| GENE_B | x.xxx | x.xxx | x.xxx |

Clinical metadata files must contain at minimum the following columns. OS.time must be in days; OS must be coded as 1 = event (death), 0 = censored. Note that the GSE84437 cohort uses pT and pN in place of composite Stage:

| sample_ID | OS.time | OS | Stage | Age | pT | pN |
|---|---|---|---|---|---|---|
| S001 | 1825 | 1 | 3 | 62 | 1 | 2 |
| S002 | 730 | 0 | 2 | 55 | 2 | 2 |

**Section 3: Data Preprocessing**

To ensure cross-platform comparability across all eight independent validation cohorts, a rigorous

multi-stage data harmonization pipeline was implemented. Each dataset was processed individually to preserve its unique biological characteristics while minimizing technical noise.

**Step 3.1: Log2-Transformation Assessment**

Prior to downstream analysis, the distribution of each expression matrix was evaluated using a quantile-based automated heuristic to determine whether log2 transformation was required. Three independent statistical criteria were assessed based on expression quantiles: (1) the 99th percentile exceeds 100; (2) the dynamic range between maximum and minimum exceeds 50 while q0.25 is positive; or (3) q0.25 lies between 0 and 1 while q0.75 lies between 1 and 2. If any criterion was met, log2 transformation was applied. Non-positive values were set to NA prior to transformation to ensure mathematical integrity.

**Step 3.2: Probe Annotation and Redundancy Resolution**

For microarray-based datasets, probes were mapped to official HGNC gene symbols using the relevant platform annotation files. Probes with ambiguous annotations (including "---", "/", or empty strings) were excluded. Where a single probe mapped to multiple loci via the "///" delimiter, only the primary symbol was retained. To resolve transcriptomic redundancy, a Maximum Value Strategy was applied: for each unique gene symbol, the probe with the highest average expression across the cohort was retained as the representative feature.

**Step 3.3: Normalization and Missing Value Management**

Between-array normalization was performed using normalizeBetweenArrays from the limma package (v3.58.1) to ensure comparability between individual samples and to eliminate within-batch technical variation. Samples with missing values in either OS.time or OS status were subsequently excluded using complete-case analysis (na.omit).

Complete preprocessing function (applied identically to all 8 validation cohorts):

```
preprocess_cohort <- function(expr_path, clinical_path) {
  library(limma)

  # Load data
  ex          <- read.csv(expr_path, row.names = 1)
  clinical <- read.csv(clinical_path)

  # Step 3.1: Log2 transformation check
  qx     <- as.numeric(quantile(ex, c(0, 0.25, 0.5, 0.75, 0.99, 1.0), na.rm = TRUE))
  LogC <- (qx[5] > 100) ||
              (qx[6] - qx[1] > 50 && qx[2] > 0) ||
              (qx[2] > 0 && qx[2] < 1 && qx[4] > 1 && qx[4] < 2)
  if (LogC) { ex[ex <= 0] <- NA; ex <- log2(ex) }

  # Step 3.2a: Remove ambiguous probe annotations (microarray platforms only)
  # anno <- read.csv("data/raw/GPL570_annotation.csv")
  # ex     <- merge(anno, ex, by = "probe_id")
  # ex     <- ex[!grepl("---|^/$|^$", ex$symbol), ]
  # ex$symbol <- sapply(strsplit(ex$symbol, "///"), `[`, 1)

  # Step 3.2b: Resolve duplicate gene symbols (max expression strategy)
```

```
    ex$symbol       <- rownames(ex)
    ex              <- aggregate(. ~ symbol, data = ex, FUN = max)
    rownames(ex) <- ex$symbol
    ex              <- ex[, -1]

    # Step 3.3a: Between-array normalization
    ex <- normalizeBetweenArrays(as.matrix(ex))

    # Step 3.3b: Remove samples with missing survival data
    common     <- intersect(colnames(ex), clinical$sample_ID)
    ex         <- ex[, common]
    clinical <- clinical[clinical$sample_ID %in% common, ]
    clinical <- na.omit(clinical)
    ex         <- ex[, clinical$sample_ID]

    return(list(expr = ex, clinical = clinical))
}

# Apply to all 8 cohorts
cohort_names <- c("ACRG_GSE62254", "TCGA_STAD", "KUCM_GSE26901",
                  "YUHS_GSE13861", "MDACC_GSE28541", "SPC_GSE15459",
                  "GSE84437", "KUGH_GSE26899")

cohorts <- list()
for (name in cohort_names) {
    cohorts[[name]] <- preprocess_cohort(
        expr_path      = paste0("data/raw/", name, "_expression.csv"),
        clinical_path = paste0("data/raw/", name, "_clinical.csv")
    )
}
```

**Section 4: Candidate Gene Identification**

To identify the most robust molecular regulators of lymph node metastasis (LNM) in GC, we implemented a rigorous four-stage filtering pipeline integrating paired-tissue transcriptomics, co-expression network modelling, and survival significance.

**Step 4.1: Paired-Tissue Differential Expression Analysis (DEA)**

Bulk RNA-seq was performed on 8 pairs of matched primary gastric tumors and metastatic lymph nodes from our institution (GEO accession: GSE246963). Raw count data were processed using the limma-voom framework. Genes with mean expression ≤ 1 across all samples were removed as a pre-filtering step. Low-abundance genes were additionally filtered using filterByExpr(). Significantly dysregulated genes were defined by |log2 fold change| > 1 and Benjamini–Hochberg adjusted $p < 0.05$, yielding 722 differentially expressed genes (384 upregulated, 338 downregulated). Both upregulated and downregulated genes were retained for downstream intersection analysis.

```
###
library(limma)
library(edgeR)

Sys.setenv(LANGUAGE = "en")
options(stringsAsFactors = FALSE)

# Load count matrix (tab-separated, genes as rows, samples as columns)
data <- read.table("data/raw/data_counts.txt", header = TRUE,
                   sep = "\t", check.names = FALSE, row.names = 1)
colnames(data) <- gsub("\\.", "-", colnames(data))

# Step 3.1: Log2 transformation check
ex <- data
qx <- as.numeric(quantile(ex, c(0, 0.25, 0.5, 0.75, 0.99, 1.0), na.rm = TRUE))
LogC <- (qx[5] > 100) ||
        (qx[6] - qx[1] > 50 && qx[2] > 0) ||
        (qx[2] > 0 && qx[2] < 1 && qx[4] > 1 && qx[4] < 2)
if (LogC) {
  ex[which(ex <= 0)] <- NA
  data <- log2(ex)
}

# Pre-filter: remove genes with mean expression <= 1
data <- data[rowMeans(data) > 1, ]

# Load sample group metadata
Control <- read.table("data/raw/tumor_metadata.txt",
                      header = FALSE, sep = "\t", check.names = FALSE)
Treat   <- read.table("data/raw/mln_metadata.txt",
                      header = FALSE, sep = "\t", check.names = FALSE)

# Set group factor and merge data by group order
group  <- factor(c(rep("Control", nrow(Control)), rep("Treatment", nrow(Treat))))
data   <- cbind(data[, Control[, 1]], data[, Treat[, 1]])
design <- model.matrix(~0 + group)
colnames(design) <- levels(group)

# limma-voom pipeline
dge  <- DGEList(counts = data, group = group)
keep <- filterByExpr(dge)
dge  <- dge[keep, , keep.lib.sizes = FALSE]
dge  <- calcNormFactors(dge)
v    <- voom(dge, design, plot = TRUE, normalize = "quantile")
```

```
fit    <- lmFit(v, design)
contrast <- makeContrasts(Treatment - Control, levels = design)
fit2 <- eBayes(contrasts.fit(fit, contrast))

# Extract all DEGs (both up- and downregulated)
allDEG    <- topTable(fit2, coef = "Treatment - Control", n = Inf)
outDiff <- allDEG[allDEG$adj.P.Val < 0.05 & abs(allDEG$logFC) > 1, ]
# Expected output: 722 DEGs (384 upregulated, 338 downregulated)

write.table(outDiff, file = "output/DEG_results.txt",
            sep = "\t", row.names = TRUE, quote = FALSE)

deg_genes <- rownames(outDiff)
```

**Step 4.2: Weighted Gene Co-expression Network Analysis (WGCNA)**

WGCNA was conducted on the ACRG training cohort (GSE62254) using the WGCNA package (v1.73) to identify gene modules co-regulated with LNM status (pN stage). The top 5,000 genes ranked by Median Absolute Deviation (MAD) were selected as input. An unsigned network was constructed using Pearson correlation, and the soft-thresholding power (β) was selected to achieve scale-free topology ($R^2 > 0.85$). Modules were detected using blockwiseModules() with a minimum module size of 30 and a merge height of 0.25. Modules significantly correlated with pN stage ($p < 0.05$) were retained, collectively comprising 2,159 genes across the blue and turquoise modules. The global random seed was fixed at 54321.

```
###
library(WGCNA)
set.seed(54321)
enableWGCNAThreads()

expr_wgcna <- t(cohorts[["ACRG_GSE62254"]]$expr)

# Select top 5,000 genes by Median Absolute Deviation (MAD)
mad_scores <- apply(expr_wgcna, 2, mad)
expr_wgcna <- expr_wgcna[, order(mad_scores, decreasing = TRUE)[1:5000]]

# Soft threshold selection (target R² > 0.85)
powers = c(c(1:10), seq(from = 12, to=20, by=2))
sft          <- pickSoftThreshold(expr_wgcna, powerVector = powers,
                                  networkType = "unsigned")
soft_power <- sft$powerEstimate
# Network construction
net <- blockwiseModules(
  expr_wgcna,
  power            = soft_power,
  networkType      = "unsigned",
```

```
  TOMType          = "unsigned",
  minModuleSize    = 30,
  mergeCutHeight = 0.25,
  numericLabels    = FALSE,
  saveTOMs         = FALSE,
  verbose          = 3
)

# Correlate modules with pN stage
pN                <- as.numeric(cohorts[["ACRG_GSE62254"]]$clinical$pN)
module_trait_cor <- cor(net$MEs, pN, use = "p")
module_trait_p    <- corPvalueStudent(module_trait_cor, nrow(expr_wgcna))
sig_modules       <- rownames(module_trait_p)[module_trait_p[, 1] < 0.05]
# Expected: blue and turquoise modules

wgcna_genes <- names(net$colors)[net$colors %in% gsub("ME", "", sig_modules)]
# Expected: 2,159 genes across significant modules
saveRDS(net, "output/WGCNA_modules.rds")
```

**Step 4.3: Gene Intersection**

To focus on genes that were both significantly dysregulated in our paired-tissue sequencing (DEA, both up- and downregulated) and co-expressed within LNM-associated WGCNA modules, we computed the logical intersection of the two gene lists, yielding 123 candidate regulators of the metastatic cascade.

```
rcommon_genes <- intersect(deg_genes, wgcna_genes)
```

**Step 4.4: Univariate Cox Regression for Survival-Based Refinement**

All 123 intersection genes were subjected to univariate Cox proportional hazards regression in the ACRG training cohort (GSE62254). Samples with missing survival time or status were excluded via na.omit. Only genes with $p < 0.05$ were retained, finalizing 91 prognostic genes as the standardized input for the machine learning framework.

```
####
library(survival)
expr_train      <- t(cohorts[["ACRG_GSE62254"]]$expr[common_genes, ])
clinical_train <- cohorts[["ACRG_GSE62254"]]$clinical

cox_results <- lapply(common_genes, function(gene) {
  df   <- data.frame(time     = clinical_train$OS.time,
                     status = clinical_train$OS,
                     expr     = expr_train[, gene])
  fit <- coxph(Surv(time, status) ~ expr, data = df)
  s    <- summary(fit)
```

```
    data.frame(gene = gene, HR = s$conf.int[1], p = s$coefficients[5])
})

cox_df                  <- do.call(rbind, cox_results)
prognostic_genes <- cox_df$gene[cox_df$p < 0.05]
# Expected output: 91 prognostic genes
write.csv(cox_df, "output/prognostic_91genes.csv", row.names = FALSE)
```

**Section 5: LMGS Construction and Risk Score Calculation**

To develop a robust and clinically deployable LMGS, we implemented an integrated machine learning framework comprising 101 distinct algorithm combinations, following the methodology of Liu et al. (Nature Communications, 2022, 13:2841). The framework was evaluated under a leave-one-out cross-validation (LOOCV) strategy across all eight cohorts to optimize for generalizability. Among all 101 combinations, RSF followed by Elastic Net (α = 0.5) achieved the highest mean C-index (0.619) and was selected as the final model.

**Step 5.1: Random Survival Forest - Feature Selection**

RSF was applied to the 91 prognostic genes in the ACRG training cohort (GSE62254) to rank features by Variable Importance (VIMP) scores generated across 1,000 survival trees. Only genes with VIMP > 0 were retained for the subsequent Elastic Net step. The global random seed was fixed at 321.

```
##
library(randomForestSRC)
set.seed(321)

train_expr <- t(cohorts[["ACRG_GSE62254"]]$expr[prognostic_genes, ])
train_clin <- cohorts[["ACRG_GSE62254"]]$clinical
train_data <- data.frame(OS.time = train_clin$OS.time,
                         OS      = train_clin$OS,
                         train_expr)

fit_rsf <- rfsrc(Surv(OS.time, OS) ~ .,
                 data       = train_data,
                 ntree      = 1000,
                 nodesize   = 10,
                 splitrule  = "logrank",
                 importance = TRUE,
                 proximity  = TRUE,
                 forest     = TRUE,
                 seed       = 321)

sel_genes <- names(which(fit_rsf$importance > 0))
```

**Step 5.2: Elastic Net (α = 0.5) — Final Model Construction**

A regularized Cox proportional hazards model was fitted on the RSF-prioritized features using Elastic Net regression (α = 0.5), which balances L1 and L2 penalties to handle correlated predictors. The optimal regularization parameter λ was determined via 10-fold cross-validation based on minimum partial likelihood deviance (λ.min = 0.03850337). Eleven genes with non-zero coefficients constituted the final LMGS panel.

```
#
library(glmnet)
set.seed(321)

train.x <- as.matrix(train_expr[, sel_genes])
train.y <- Surv(train_data$OS.time, train_data$OS)

fit_cv      <- cv.glmnet(train.x, train.y, family = "cox",
                         alpha = 0.5, nfolds = 10)
# Expected: fit_cv$lambda.min = 0.03850337

glmnet_fit <- glmnet(train.x, train.y, family = "cox",
                     nfolds = 10, keep = TRUE,
                     alpha   = 0.5, lambda = fit_cv$lambda.min)

coef_matrix <- coef(fit_cv, s = "lambda.min")
# Expected non-zero genes: SH3RF2, CASP7, PLEKHS1, HADH, CYC1, GFPT1,
#                          C15orf48, DES, RORA, ST3GAL1, ACTG2

lmgs_table <- data.frame(
  gene        = rownames(coef_matrix)[coef_matrix[, 1] != 0],
  coefficient = coef_matrix[coef_matrix[, 1] != 0, 1]
)
write.csv(lmgs_table, "output/LMGS_11genes_coefficients.csv")
```

**Step 5.3: Risk Score Calculation and Fixed Cutoff Validation**

Risk scores were calculated for all cohorts using the predict() function with type = "link", which computes the linear predictor (i.e., the weighted sum of gene expression values multiplied by their respective coefficients). The fixed cutoff (median risk score of the ACRG training cohort = −0.0649505) was established from the training cohort and applied uniformly to all validation cohorts, ensuring clinical transferability and preventing data leakage.

```
# --- Training cohort (ACRG_GSE62254) risk score ---
risk_scores_train <- predict(glmnet_fit, newx = train.x,
                             s = fit_cv$lambda.min, type = "link")
risk_scores_train      <- as.data.frame(risk_scores_train)
colnames(risk_scores_train)[colnames(risk_scores_train) == "1"] <- "score"
train_data$risk_score  <- risk_scores_train$score
```

```
# Fixed cutoff: median of training cohort risk scores
fixed_cutoff                    <- median(train_data$risk_score)
# Expected value: -0.0649505
train_data$risk_group       <- ifelse(train_data$risk_score > fixed_cutoff, "High", "Low")

# --- Validation cohorts: loop using predict() ---
all_results                     <- list()
all_results[["ACRG_GSE62254"]] <- train_data

for (name in cohort_names[cohort_names != "ACRG_GSE62254"]) {
  clin_val <- cohorts[[name]]$clinical
  test.x     <- as.matrix(t(cohorts[[name]]$expr[sel_genes, ]))

  rs <- predict(glmnet_fit, newx = test.x,
                     s = fit_cv$lambda.min, type = "link")
  rs <- as.data.frame(rs)
  colnames(rs)[colnames(rs) == "1"] <- "score"

  clin_val$risk_score <- rs$score
  clin_val$risk_group <- ifelse(rs$score > fixed_cutoff, "High", "Low")
  all_results[[name]] <- clin_val
}

# Save all risk scores
all_df <- do.call(rbind, lapply(names(all_results), function(n) {
  df <- all_results[[n]]; df$cohort <- n; df
}))
write.csv(all_df, "output/risk_scores_all_cohorts.csv", row.names = FALSE)
```

For independent verification without requiring the original model object, the finalized 11-gene coefficients are additionally provided below in hard-coded form. This approach computes a mathematically identical risk score via direct matrix multiplication (Score = Σ Coefficient × Expression) and can be applied by any independent group without re-running the training pipeline.

```
# Hard-coded finalized 11-gene weights (identical to glmnet_fit coefficients)
lmgs_weights <- c(
  "SH3RF2"     = -0.844743431,
  "CASP7"       = -0.683513762,
  "PLEKHS1"   = -0.235704642,
  "HADH"         = -0.154002149,
  "CYC1"         = -0.110519150,
  "GFPT1"       = -0.109081726,
  "C15orf48" = -0.005218872,
```

```r
  "DES"       =  0.091642594,
  "RORA"      =  0.195787587,
  "ST3GAL1"   =  0.203923744,
  "ACTG2"     =  0.205650600
)

# Hard-coded fixed cutoff (median risk score of ACRG_GSE62254 training cohort)
fixed_cutoff <- -0.0649505

# Direct matrix multiplication — mathematically equivalent to predict() above
for (name in cohort_names) {
  expr_val <- t(cohorts[[name]]$expr[names(lmgs_weights), ])
  clin_val <- cohorts[[name]]$clinical

  # Score = Expression %*% Weights
  rs <- as.numeric(as.matrix(expr_val) %*% lmgs_weights)

  clin_val$risk_score <- rs
  clin_val$risk_group <- ifelse(rs > fixed_cutoff, "High", "Low")
  all_results[[name]] <- clin_val
}
```

**Section 6: Model Evaluation and Key Figure Reproduction**

All evaluation analyses operate directly on the all_results object generated in Section 5.

**Step 6.1: Kaplan–Meier Survival Curves**

```r
#R
library(survival); library(survminer)

for (name in cohort_names) {
  df      <- all_results[[name]]
  km_fit <- survfit(Surv(OS.time, OS) ~ risk_group, data = df)
  p <- ggsurvplot(km_fit, data = df,
                  pval        = TRUE,
                  risk.table = FALSE,
                  palette     = c("firebrick", "skyblue"),
                  title       = name)
  pdf(paste0("output/figures/Figure_KM_", name, ".pdf"),
      width = 6, height = 5)
  print(p)
  dev.off()
}
```

**Step 6.2: Time-Dependent ROC Curves**

```r
#
```

```
library(timeROC)

for (name in cohort_names) {
  df       <- all_results[[name]]
  roc_res <- timeROC(T        = df$OS.time,
                     delta  = df$OS,
                     marker = df$risk_score,
                     cause  = 1,
                     times  = c(365, 1095, 1825),
                     iid    = TRUE)
  cat(name, "| AUC 1y:", roc_res$AUC[1],
          "3y:", roc_res$AUC[2],
          "5y:", roc_res$AUC[3], "\n")
}
```

**Step 6.3: Calibration Curves**

Calibration curves were generated using the rms package (v6.8.1) to assess agreement between LMGS-predicted survival probabilities and actual observed outcomes at 1, 3, and 5 years. Bootstrap validation was applied with B = 100 iterations. The grouping parameter m was set to one-third of each cohort's sample size (ceiling(nrow(data) / 3)).

```
#R
library(rms); library(survival)

for (name in cohort_names) {
  data   <- all_results[[name]]
  m_val <- ceiling(nrow(data) / 3)

  os       <- as.numeric(data$OS.time)
  censor <- as.numeric(data$OS)
  S        <- Surv(os, censor)

  ddist <- datadist(data$risk_score)
  options(datadist = "ddist")

  factor <- "risk_score"
  f_form <- as.formula(paste("S", factor, sep = "~"))

  pdf(paste0("output/figures/Figure_Calibration_", name, ".pdf"),
      width = 6, height = 6)

  # 1-year calibration
  coxm <- cph(f_form, x = TRUE, y = TRUE, data = data,
              surv = TRUE, time.inc = 365)
```

```
  cal   <- calibrate(coxm, cmethod = "KM", method = "boot",
                     u = 365, m = m_val, B = 100)
  plot(cal, xlim = c(0, 1), ylim = c(0, 1), col = "red",
       main = paste("Calibration —", name))

  # 3-year calibration
  coxm <- cph(f_form, x = TRUE, y = TRUE, data = data,
              surv = TRUE, time.inc = 1095)
  cal   <- calibrate(coxm, cmethod = "KM", method = "boot",
                     u = 1095, m = m_val, B = 100)
  par(new = TRUE)
  plot(cal, xlim = c(0, 1), ylim = c(0, 1),
       xlab = "", ylab = "", xaxt = "n", yaxt = "n", col = "orange")

  # 5-year calibration
  coxm <- cph(f_form, x = TRUE, y = TRUE, data = data,
              surv = TRUE, time.inc = 1825)
  cal   <- calibrate(coxm, cmethod = "KM", method = "boot",
                     u = 1825, m = m_val, B = 100)
  par(new = TRUE)
  plot(cal, xlim = c(0, 1), ylim = c(0, 1),
       xlab = "", ylab = "", xaxt = "n", yaxt = "n", col = "limegreen")

  legend("topleft", legend = c("1-year", "3-year", "5-year"),
         col = c("red", "orange", "limegreen"), lty = 1, bty = "n")
  dev.off()
}
```

**Step 6.4: Decision Curve Analysis**

Decision curves were generated using the ggDCA package (v1.2) to quantify the incremental clinical net benefit of the LMGS relative to staging variables alone and to a combined model. Note that the GSE84437 cohort does not contain composite AJCC Stage and instead uses pT and pN as separate variables; the staging model for this cohort is therefore constructed accordingly.

```
#R
library(rms); library(ggDCA); library(survival)
for (name in cohort_names) {
  data <- all_results[[name]]

  options(datadist = NULL)
  dd <- datadist(data)
  options(datadist = "dd")

  # GSE84437 uses pT + pN instead of composite Stage
  if (name == "GSE84437") {
```

```
    Stage_mod <- cph(Surv(OS.time, OS) ~ pT + pN,
                                data = data, x = TRUE, y = TRUE, surv = TRUE)
    Combined   <- cph(Surv(OS.time, OS) ~ risk_score + pT + pN,
                                data = data, x = TRUE, y = TRUE, surv = TRUE)
  } else {
    Stage_mod <- cph(Surv(OS.time, OS) ~ Stage,
                                data = data, x = TRUE, y = TRUE, surv = TRUE)
    Combined   <- cph(Surv(OS.time, OS) ~ risk_score + Stage,
                                data = data, x = TRUE, y = TRUE, surv = TRUE)
  }

  LMGS_mod <- cph(Surv(OS.time, OS) ~ risk_score,
                            data = data, x = TRUE, y = TRUE, surv = TRUE)

  dca_all <- dca(Stage_mod, LMGS_mod, Combined,
                          times = c(365, 1095, 1825))

  p <- ggplot(dca_all) +
    facet_wrap(~time) +
    theme_bw() +
    labs(title = paste("DCA —", name))

  ggsave(paste0("output/figures/Figure_DCA_", name, ".pdf"),
              p, width = 9, height = 4)
}
```

**Concluding Statement on Reproducibility**

This document provides a fully self-contained, end-to-end computational pipeline sufficient for any independent group to reproduce all primary findings reported in this study. Key reproducibility safeguards include: (1) a clearly defined input file structure with explicit GEO accession numbers and file naming conventions (Section 0); (2) a complete software environment specification with exact package versions and installation commands (Section 1); (3) standardized input data format requirements (Section 2); (4) fully executable preprocessing, gene identification, and model construction code with exact parameters and random seeds throughout (Sections 3–5); (5) both the original predict()-based risk score calculation and a hard-coded coefficient implementation to facilitate independent verification without requiring the original model object (Section 5.3); and (6) main figure reproduction commands for all primary and supplementary figures (Section 6).

For further technical enquiries or raw data requests, please contact the corresponding author.
